# DNA engineered micromotors powered by metal nanoparticles for motion based cellphone diagnostics

**DOI:** 10.1038/s41467-018-06727-8

**Published:** 2018-10-16

**Authors:** Mohamed Shehata Draz, Kamyar Mehrabi Kochehbyoki, Anish Vasan, Dheerendranath Battalapalli, Aparna Sreeram, Manoj Kumar Kanakasabapathy, Shantanu Kallakuri, Athe Tsibris, Daniel R. Kuritzkes, Hadi Shafiee

**Affiliations:** 1 Division of Engineering in Medicine, Department of Medicine, Brigham and Women’s Hospital, Harvard Medical School, Boston, 02139 MA USA; 2 Department of Medicine, Harvard Medical School, Boston, 02115 MA USA; 3 Faculty of Science, Tanta University, Tanta, 31527 Egypt; 4 Division of Infectious Diseases, Brigham and Women’s Hospital, Harvard Medical School, Boston, 02139 MA USA

## Abstract

HIV-1 infection is a major health threat in both developed and developing countries. The integration of mobile health approaches and bioengineered catalytic motors can allow the development of sensitive and portable technologies for HIV-1 management. Here, we report a platform that integrates cellphone-based optical sensing, loop-mediated isothermal DNA amplification and micromotor motion for molecular detection of HIV-1. The presence of HIV-1 RNA in a sample results in the formation of large-sized amplicons that reduce the motion of motors. The change in the motors motion can be accurately measured using a cellphone system as the biomarker for target nucleic acid detection. The presented platform allows the qualitative detection of HIV-1 (n = 54) with 99.1% specificity and 94.6% sensitivity at a clinically relevant threshold value of 1000 virus particles/ml. The cellphone system has the potential to enable the development of rapid and low-cost diagnostics for viruses and other infectious diseases.

## Abstract (web summary)

Micromotors have a range of potential healthcare applications. Here, the authors describe the development of a metal nanoparticle DNA micromotor which can be used to detect human HIV-1 by a change in the motion of the micromotors, monitored by cell phone camera, triggered by binding of HIV-1 RNA.

## Introduction

The timely management of infectious diseases especially in the developing countries where there are limited laboratory infrastructure and trained staff is a major global health-care challenge^1–3^. Diagnostic platforms that meet the World Health Organization’s ASSURED criteria (Affordable, Sensitive, Specific, User friendly, Rapid, Equipment-free, and Delivered) with the ability to be seamlessly integrated with appropriate and effective surveillance mechanisms can shift the paradigm in outbreak control and the prevention of new epidemics^3–5^. Of particular interest is early human immunodeficiency virus (HIV) detection, timely initiation of antiretroviral therapy (ART), and monitoring ART for efficient management of HIV infection particularly in resource-poor settings where >70% of the infected individuals live^6^.

The rapid advances in consumer electronics and portable communication systems particularly  cellphones have led to faster and cheaper approaches of data acquisition and significant growth of cellphone subscribers worldwide particularly in the developing countries^7–10^. The global cellphone adoption and cellphone adoption in just Africa has jumped to almost 4.8 billion and 0.5 billion, respectively in 2016 and will reach to 5.7 billion and 0.725 billion by 2020, respectively^8–10^. Such significant cellphone adoption particularly in resource-poor settings combined with its powerful computing ability and built-in sensors present a promising potential in developing mobile health (mhealth) tools and surveillance diagnostics that can help us in closing the gap in infectious disease management through providing appropriate diagnostic solutions^11–13^. The advances in telecommunications combined with the recent changes in consumer behavior on wearable sensors and self-testing have led to the development of a series of mhealth diagnostic and surveillance technologies for disease detection and infection management^14–21^.

Recent progress in material synthesis and engineering can allow seamless integration with cellphone systems to develop advanced digital health tools and technologies. Self-propelling nanoparticles (NPs)/microparticles are among the most exciting materials that possess unique properties and have a tremendous potential for disease diagnosis and treatment monitoring^22–24^. These materials are readily synthesized in different shapes (e.g., spherical, rod, wire, tube, and polymer aggregates) and catalytic, magnetic, and acoustic forces can power their motion. Catalytically powered motors are usually designed to harness specific catalytic reactions either through using enzymes or transition metals, such as platinum, silver, gold, copper, nickel, and iron. Simple integration with portable devices and stability over time especially compared to fluorescent-based sensing (i.e., resistant to photobleaching, temperature, and pH)^25–27^ are some of the advantages of catalytic motion-based sensing approaches. Furthermore, motion-based optical sensing does not require bulky optical components or expensive equipment usually used for fluorescent-based microscopy. It can also allow rapid testing compared to the conventional nucleic acid detection methods, such as polymerase chain reaction (PCR) and enzyme-linked immunosorbent assay (ELISA)^25,27,28^. So far, catalytic motor systems have been reported for DNA and protein sensing and diagnosis of different pathogens and diseases^29–38^.

**Fig. 1:**
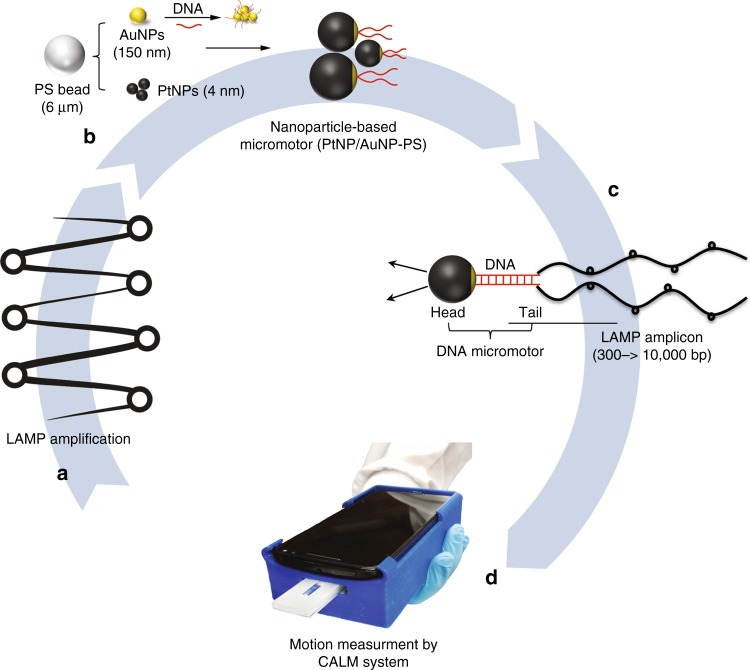
Schematic presentation of HIV-1 detection using the cellphone system. The developed system integrates cellphone-based optical sensing, loop-mediated isothermal amplification and micromotor motion (CALM). **a** A loop-mediated isothermal amplification (LAMP) reaction is performed to amplify the nucleic acid of HIV-1 and large-size looped amplicons. **b** The formed amplicons are mixed with DNA-modified micromotors that are specifically designed using 6-μm polystyrene (PS) beads covered with a hybrid surface layer of platinum (Pt) and gold (Au) nanoparticles to power the catalytic motion of motors in the presence of hydrogen peroxide. **c** The capture of LAMP amplicons on the surface of motors results in the formation of motile assemblies with a catalytic head of motors and large tail of DNA. **d** The motion of these assemblies can be detected and measured using a cellphone system on-chip for qualitative HIV-1 detection

Here, we present a cellphone-based assay for HIV-1 molecular detection using loop-mediated isothermal amplification (LAMP) and micromotors (CALM). The large stem-looped amplicons formed through LAMP amplification are uniquely adopted to change the motion of specifically DNA-engineered micromotors powered by metal NPs (i.e., platinum nanoparticles (PtNPs) and gold nanoparticles (AuNPs)) indicating the presence of the HIV-1 using the cellphone system (Fig. 1). The efficiency of the CALM system in qualitative detection of HIV-1 is evaluated using phosphate-buffered saline (PBS) and serum samples (*n* = 54) spiked with different concentrations of HIV-1 above and below a clinically relevant threshold value of 1000 virus particles/ml. The cellphone system is also tested using HIV-infected patient plasma samples (*n* = 6) by comparing the results of the motor-based sensing with the results obtained by a quantitative real-time PCR assay targeting a highly conserved region of integrase in the HIV-1 *pol* gene with single-copy sensitivity (iSCA).

## Results

### Platinum motor preparation and characterization

**Fig. 2:**
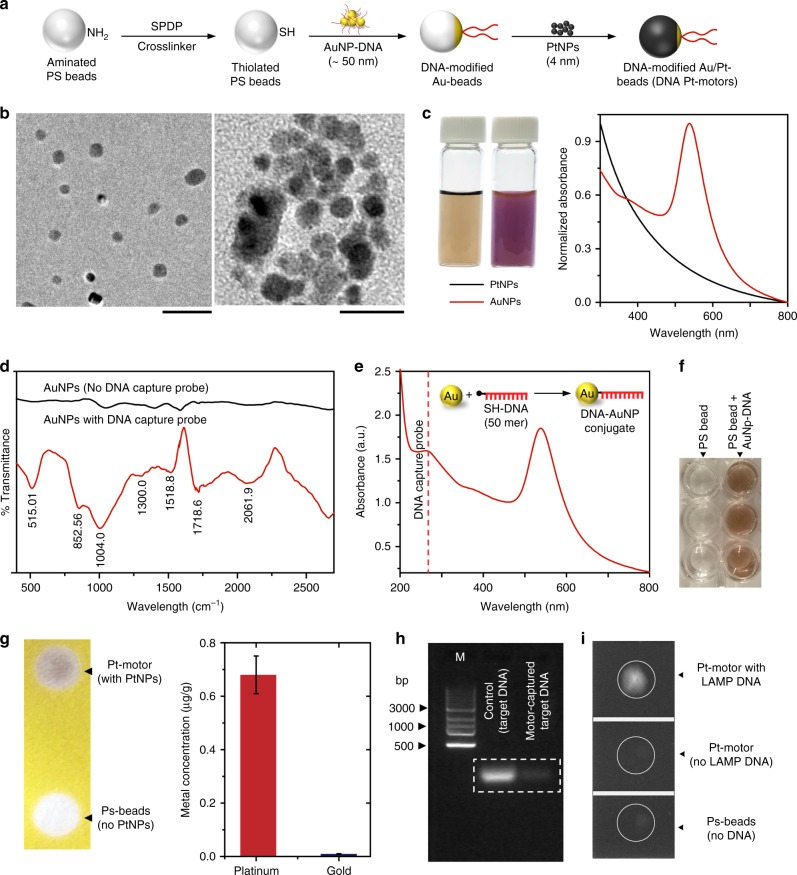
Pt-motor preparation and characterization. **a** Schematic of motor preparation reaction. Polystyrene (PS) beads that carry amine groups on its surface are initially activated with excess of succinimidyl 3-(2-pyridyldithio)propionate (SPDP) crosslinker to prepare thiolated (-SH) beads. Then gold nanoparticles (AuNPs) modified with DNA capture probe (30-mer in size) and platinum nanoparticles (PtNPs) are added to the surface of thiolated beads following the well-known thiol–metal chemistry. **b** Transmission electron micrographs for the prepared AuNPs (left, scale bar = 200 nm) and PtNPs (right, scale bar = 10 nm). **c** Digital images of PtNPs (brown in color) and AuNPs (red in color) solutions used in micromotor preparation and the corresponding UV-vis absorbance spectra of  the as-prepared nanoparticle solutions. **d** FT-IR analysis of AuNP-modified DNA capture probe. **e** UV-vis absorbance spectrum of AuNPs modified with DNA capture probe, showing an absorbance peak at 260 nm that is characteristic to DNA. **f** Silver staining reaction results confirming the stable addition of AuNPs–DNA to the surface of PS beads for motor preparation. **g** Digital image of DNA–AuNP beads after addition of PtNPs, the brown color indicates the heavy accumulation of PtNPs on the surface of beads followed by the results of ICP-MS analysis confirming the presence of Pt and Au metal on the surface of the prepared micromotors (DNA Pt/Au-beads). Error bars represent the standard deviation of three independent experiments. **h** Agarose gel electrophoresis confirming the presence of DNA capture probe on the surface of the prepared motors using a synthetic target DNA. Lane M: 1-kb DNA ladder marker. The dashed white line indicates the DNA bands of both the target DNA captured and isolated by motors and control of 1 μM target DNA (no capture by motors). **i** Fluorescence imaging of LAMP amplicons captured and isolated using the prepared motors confirming the full activity of the motors to specifically interact with the target DNA amplicons. The white circle highlights the position of casted Pt-motor samples on the surface of the imaged glass slide

The micromotors used in this study are PtNP-coated spherical polystyrene (PS) beads (with density of 1.04 g/cm³) indirectly engineered with short DNA probes through a middle piece of spherical AuNP (Fig. 2a). A detailed schematic for synthesis of Pt-motors is shown in Supplementary Fig. 1. The motor preparation reaction includes the direct coupling of AuNPs and PtNPs to the surface of amine-functionalized PS beads using a heterobifunctional crosslinker of succinimidyl 3-(2-pyridyldithio)propionate (SPDP). The beads were initially activated with SPDP-forming thiolated beads to allow the thiol–metal-based coupling with NPs (i.e., PtNPs and AuNPs). Prior to coupling reaction, AuNPs were modified with thiolated DNA probes of 30-mer oligonucleotides that specifically target HIV-1 *gag*. The prepared AuNP-DNA conjugates were mixed with the SPDP-activated beads with a molar ratio of 1:10 to minimize the number of DNA probes on the surface of beads. The remaining surface of PS beads was coated by adding excess amount of PtNPs (Fig. 2a).

Transmission electron microscopy (TEM) of the synthesized NPs showed that both the synthesized AuNPs and PtNPs were spherical in shape with diameters of 57.721 ± 5.181 nm (data reported as mean ± standard deviation) and 3.43 ± 1.336 nm, respectively (Fig. 2b). Digital images and ultraviolet–visible (UV-vis) spectroscopic analysis results for AuNPs and PtNPs are shown in Fig. 2c and Supplementary Fig. 2. In addition, dynamic light scattering (DLS) analysis results confirmed the stability of the prepared AuNPs and PtNPs with polydispersity index values <0.45 (Supplementary Fig. 3, 4) and zeta potential values of −14 to −29 mV (Supplementary Fig. 5, 6). The conjugation of DNA to AuNPs was confirmed using UV-vis spectroscopy and Fourier-transform infrared spectroscopy (FT-IR) techniques. Figure 2d shows the FT-IR spectra of non-modified prepared AuNPs and AuNPs conjugated with DNA probes. The addition of DNA resulted into a group of peaks around 515.01, 852.56, 1004.0, 1300.0, 1518.8, 1718.6, and 2061.9/cm that are specific for NH_2_ and pyridine of DNA nucleotides^39^. In addition, the number of DNA capture probes per NP was quantified using UV-vis spectroscopy (Fig. 2e and Supplementary Methods). The results indicated that the average ratio of DNA/AuNPs was 12.1 ± 0.12 DNA probe molecule per each AuNP. The efficiency of AuNP–DNA conjugates coupling to the surface of PS beads was evaluated using silver staining technique. The presence of DNA–AuNPs on the surface of beads induced a rapid change of the silver staining reaction into a dense dark brown color compared with control samples where non-modified PS beads were added (no AuNPs) (Fig. 2f). The stability and reactivity of the fully structured motors (PtNP/AuNP–DNA-modified PS) were confirmed using inductive couple plasma–mass spectroscopy (ICP-MS) and gel electrophoresis techniques (Fig. 2g–i). ICP-MS analysis showed that each PS bead is modified with an average number of 1.335 ± 0.9161 and 386,044.1 ± 10.9161 of AuNPs and PtNPs, respectively (Supplementary Methods). In addition, the deposition of PtNPs with its characteristic intense brown color on the surface of PS beads was easily observed as a visible brown color when the prepared motor solution was dropcasted on a sheet of chromatography paper, confirming the heavy surface modification of beads with PtNPs (Fig. 2g). On the other hand, agarose gel electrophoresis technique was applied to test the efficiency of the fully structured motors (PtNP/AuNP–DNA-modified PS beads) in capturing synthetic target DNA (Fig. 2h). Synthetic target DNA was mixed and allowed to hybridize to DNA capture probes present on the surface of Pt-motor. The hybridization reaction products of motors and target DNA were then isolated by centrifugation, washed, and the captured synthetic target DNA molecules were released by incubation at 95 °C for 5 min The released target DNA was then tested using agarose gel electrophoresis technique. The results showed a clear band at 180 bp that is specific for the synthetic target DNA captured and isolated using the prepared Pt-motors (Fig. 2h). Furthermore, fluorescence spectroscopy indicated a 30% capture efficiency of LAMP amplicons on the surface of motors (Fig. 2i and Supplementary Methods).

### Platinum motor motion testing and optimization

**Fig. 3:**
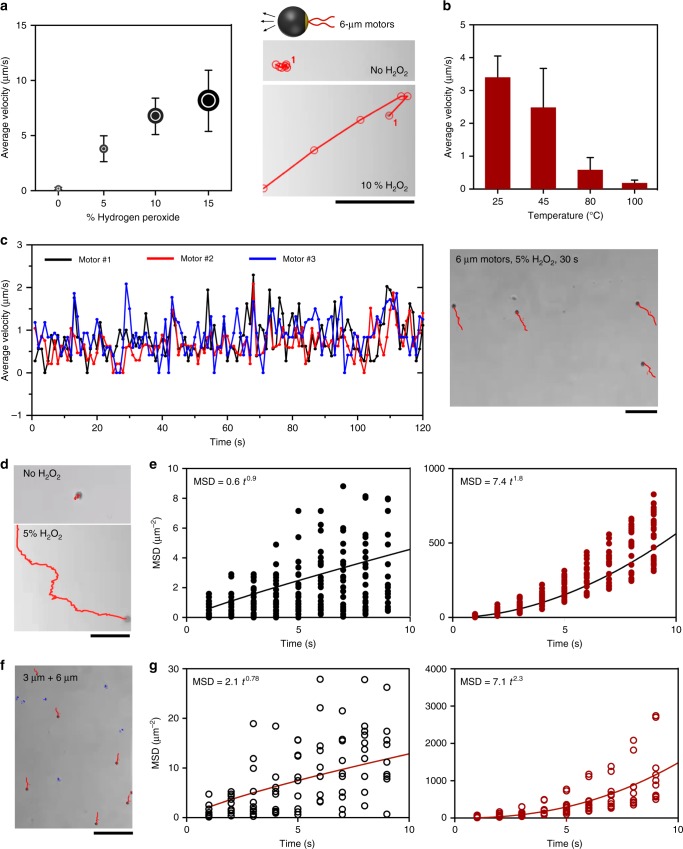
Micromotor motion. **a** Motion of 6 μm motors in solutions with different concentrations of hydrogen peroxide (H_2_O_2_). The digital images show motion trajectories of the prepared 6 μm motors in the presence of 0% H_2_O_2_ solution and 10% H_2_O_2_ solution (scale bar = 50 μm). **b** Thermal stability of motors at different temperatures tested in 5% H_2_O_2_ solution. **c** Stability of the motor motion with time (120 s) and the digital image show the motion trajectories of 6-μm motors in 15% H_2_O_2_ solution after 30 s (scale bar = 50 μm). **d** Motion trajectories of 6-μm motors with and without H_2_O_2_ (scale bar = 10 μm). **e** Mean squared displacement (MSD) plotted against time (*t*) for motor motion in 0% H_2_O_2_ (no H_2_O_2_, black dots) or 5% H_2_O_2_ (red dots). The black lines indicate the average slope derived from all individual particle trajectories (*n* = 20). **f** Motion trajectories of a mixture of 3-μm beads (no PtNPs or AuNPs were added, with blue trajectories) and 6-μm motors (beads with PtNPs and AuNPs, with red trajectories). Scale bar is 100 μm. **g** MSD analysis of a mixture of 3-μm beads (no PtNPs or AuNPs were added, black circles) and 6-μm motors (beads with PtNPs and AuNPs, red circles). The red lines indicate the average slope derived from all individual particle trajectories (*n* = 20). Error bars represent the standard deviation of at least three independent experiments

The velocity of Pt-motors prepared from 6-μm beads was tested in the presence and absence of H_2_O_2_. Figure 3a shows the effect of the concentration of H_2_O_2_ on the motion of 6-μm Pt-motors. In the absence of H_2_O_2_, motors were just vibrating due to the Brownian motion, and in the presence of H_2_O_2_, the average velocity of the motors increased at a rate of ~0.7 μm/s for 1% increase of H_2_O_2_ concentration (Supplementary Fig. 7 and Supplementary Movie 1). Our sensing protocol relies on monitoring the change in motor motion due to the LAMP amplicon reaction on the surface of motors using DNA capture probes through thermal hybridization. Thus it was necessary to test the effect of temperature and incubation time in H_2_O_2_ on the velocity of the prepared motors. We incubated aliquots of the prepared motors at 45, 80, and 100 °C for 10 min. The results indicated that the velocity of the prepared motors decreased with the increase in the temperature and there was a 10% loss of motion at 45 °C when compared to control (incubated at 25 °C as room temperature) (Fig. 3b, Supplementary Fig. 8). On the other hand, the prepared motors were stable in their motion with time of incubation in H_2_O_2_. Figure 3c presents the motion of motors for 120 s in 5% H_2_O_2_ solution. In the presence of H_2_O_2_, the motors autonomously move in a self-propelled fashion that is in principle due to the consumption of H_2_O_2_ and generation of gas bubbles. To investigate the motion of the prepared Pt-motors, their motion trajectories in the presence and absence of H_2_O_2_ were recorded under a bright-filed light microscope and then analyzed by plotting the mean squared displacement (MSD) against time (*t*). MSD is known to be proportional to *t*^*α*^ for scaling exponent *α*^40^. We found that the MSD versus time had a near linear dependence (*α* = 0.9) when no H_2_O_2_ was added, as is the case for random diffusion (*α* = 1), while in the presence of 5% H_2_O_2_ solution, *α* = 1.8 indicating that the motor motion is caused by the catalytic activity of the surface PtNPs and differs from random diffusion (Fig. 3d, e). To further confirm the catalytic nature of the prepared motors (i.e., move due to the decomposition of H_2_O_2_ by PtNPs), samples with a mixture of 6-μm motor (coated with PtNPs) and non-modified 3-μm beads (no PtNPs) in 5% H_2_O_2_ solution were tested and their motion was analyzed using bright-field light microscopy and Image J software. The slope of MSD plot of the 3-µm beads and 6-µm Pt-motors suggests a fundamentally different mode of motion (Fig. 3f, g and Supplementary Movie 2).

### Development of Pt-motor tracking cellphone system

**Fig. 4:**
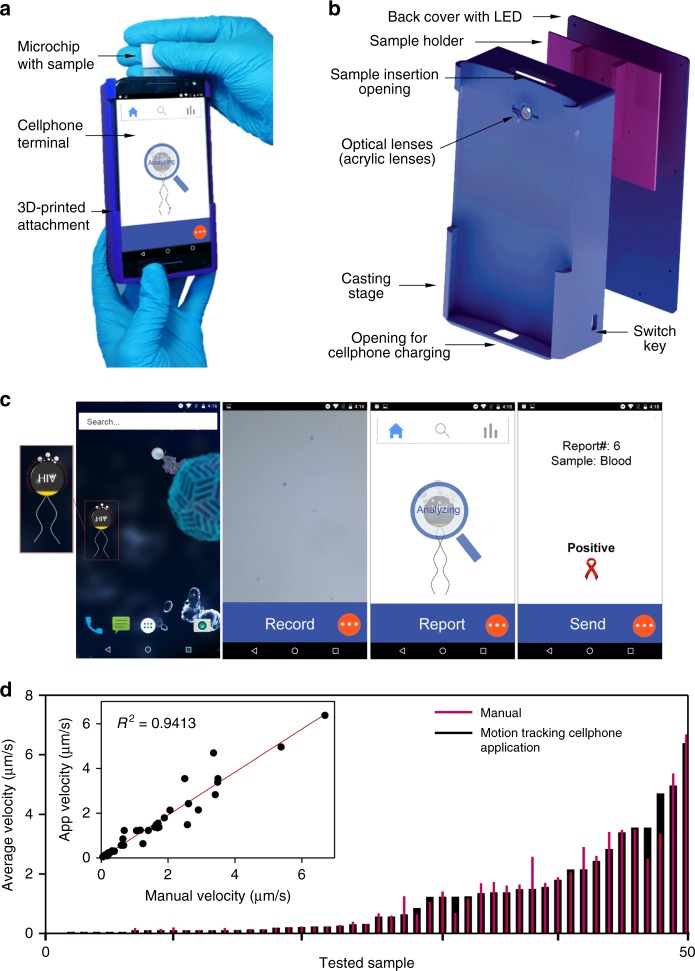
Design of the CALM system. **a** Operating CALM system with a cellphone optical accessory and a disposable microchip. **b** Exploded 3D schematic of the cellphone attachment. The main components of the cellphone attachment including a casting stage with optics, sample holder, and back cover with LED was printed using PLA. **c** Motion tracking application used to detect and measure the motion of DNA-motors in H_2_O_2_ solution. **d** Correlation between the performance of the motion tracking application and bright-field light microscopy coupled with the ImageJ software in velocity detection of different motor samples (*n* = 50). *R*^2^ is the coefficient of determination of the linear regression analysis

The cellphone system used in visualizing and tracking the motion of micromotors comprises an android terminal (XT1575, Motorola) modified with an optical attachment and a cellphone application on a single-channel microfluidic device (Fig. 4a). The microchip used to test the motion of motors was prepared of poly(methyl methacrylate) (PMMA) substrate and a glass slide attached to each other using a 80-μm double-sided adhesive (DSA) machined with a laser cutter to fabricate a single microchannel with 2 mm width (Supplementary Fig. 9). The cellphone attachment was designed in Solidworks and three-dimensional (3D) printed with low-cost polylactic acid (PLA) material. The 3D printed enclosure housed a broadband white light-emitting diode (LED), a 3.3-V battery, a switch, and inexpensive optical lenses. The 3D construct also includes a sample holder to focus the sample between the two lenses and the cellphone camera (Fig. 4b). The optical attachment and sample holder were custom-designed to facilitate chip insertion and positioning on the attachment through a simple slide-on mechanism and in a way that the chip remains in optimal focus without the need for manual focusing. The software on the cellphone was developed in Android Studio using OpenCV (ver. 3.1.0) libraries with a user-friendly interface to guide the user through the testing process (Fig. 4c). The cellphone application records videos of samples, enumerates motors, automatically calculates the velocity of motors, and reports the results in <1 min. The developed application was able to record sample videos at a rate of 30 frames per second (fps) with a maximum effective field of view of 320 × 240 pixels for the cellphone used in this study (Motorola MotoX). The developed cellphone system was first calibrated using a micrometer scale shown in Supplementary Fig. 10. The performance of the developed system in visualizing, counting, and tracking the motion of Pt-motors was tested and correlated to the manual counts using bright-field microscopy. The results indicated a correlation coefficient of 0.9413 with a standard error of 0.3592 and a correlation coefficient of 0.9028 with a standard error of 0.3121 for motor velocity and enumeration, respectively. Furthermore, there was no statistical difference between the measurements (*n* = 50) from the motion tracking application and the manual count performed by bright-field microscopy (*P* > 0.05, paired *t* test) and with 95% confidence interval (CI) of −0.1564 to 0.04814 and −0.1491 to 0.02913 for motor velocity and enumeration, respectively (Fig. 4d, Supplementary Fig. 11 and Supplementary Table 1).

### LAMP reaction and validation of the CALM system

**Fig. 5:**
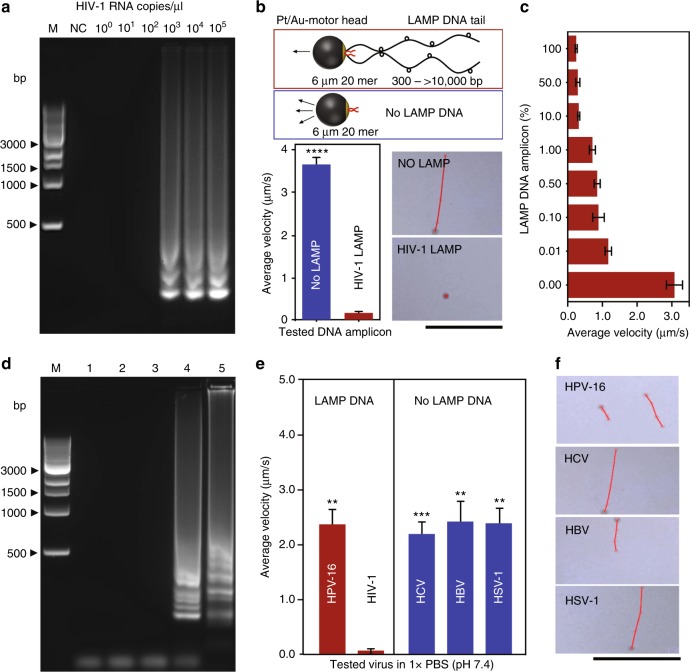
Validation of the CALM system using HIV-1 LAMP amplicon. Detection sensitivity: **a** agarose gel electrophoresis image of serially diluted HIV-1 RNA samples (0 copies/μl–10^5^ copies/μl). Lane M: 1-kb DNA ladder marker; Lane NC: negative control (without target RNA template); **b** average velocity of motors (*n* = 30) with (in red box) and without (in blue box) HIV-1 LAMP amplicons generated from HIV-1 RNA concentration of 10^4^ copies/μl. The digital images show motion trajectories of motors in the presence and absence of LAMP amplicons tested in 5% H_2_O_2_ (scale bar = 100 μm); **c** average velocity of motors in the presence of 0% (no LAMP, control) to 100% dilutions of HIV-1 LAMP amplification products    (10^4^ copies/μl) prepared in LAMP reaction buffer. Detection specificity: **d** agarose gel electrophoresis image of HIV-1 and human papillomavirus 16 (HPV-16) and different non-targeted viruses, including hepatitis C virus (HCV), hepatitis B virus (HBV), and herpes simplex virus type-1 (HSV-1); Lane M: 1-kb DNA ladder marker; Lane 1: HSV-1; Lane2: HBV; Lane 3: HCV; Lane 4: HIV-1; Lane 5: HPV-16; **e** average velocity of motors (*n* = 30) in the presence of the amplification products of the target and non-target viruses; **f** representative digital images show the motion trajectories of motors in the presence of LAMP amplification products generated with the target and non-target viruses (scale bar = 100 μm). The concentration of the nucleic acid of all of the tested viruses was adjusted to 10^4^ copies/μl before LAMP amplification. The results are expressed as the average values of three independent experiments. Error bars represent standard deviations. **P < 0.01, ***P < 0.001, ****P < 0.0001 versus the corresponding group with the target HIV-1, calculated using unpaired *t* test

Reverse transcription-loop mediated isothermal amplification (RT-LAMP) was performed using a set of four primers that target *gag* gene of HIV-1 (Table 1 and Supplementary Fig. 12) following the standard protocol^41,42^. Different concentrations of HIV-1 RNA template were prepared and used as a target in LAMP reaction. The amplification product was characterized using agarose gel electrophoresis (Fig. 5a). The results indicated the formation of large-sized DNA amplicons appeared as ladder-like patterns with many bands (>320 bp in size) and the amount of these amplicons was proportional to the concentration of HIV-1 RNA.

**Table 1:** List of DNA sequences used in this study

Oligonucleotide	Sequence
LAMP F3 primer	5′-GGTAAGAGATCAGGCTGAACATC-3′
LAMP B3 primer	5′-GCTGGTCCTTTCCAAAGTGG-3′
LAMP FIP primer	5′-CCCCAATCCCCCCTTTTCTTAGACAGCAGTACAAATGGCA-3′
LAMP BIP primer	5′-AGTGCAGGGGAAAGAATAGTAGACCTGCTGTCCCTGTAATAAACCC-3′
Pt-motor capture probe	5′-TTAAGACAGCAGTACAAATGGCAGTAAAAA/3ThioMC3-D/-3′
Pt-motor capture target DNA	5′-TTTTCTTTTAAAATTGTGGATGAATACTGCCATTTGTACTGCTGTCTTAA-3′

To detect the motion of motors in the presence and absence of HIV-1 LAMP amplicons, the motors were mixed with LAMP amplicons and allowed to hybridize at 80 °C (Supplementary Methods). The formed motor–LAMP DNA assemblies were tested in 5% H_2_O_2_ solution. In the presence of HIV-1 LAMP amplicons, the velocity of the motors (*n* = 30) was significantly (*P* < 0.0001, unpaired *t* test) decreased by 95.26% compared to control where no HIV-1 LAMP amplicons were added (only Pt-motors) (Fig. 5b, Supplementary Fig. 13 and Supplementary Movie 3, 4). Subsequently, different dilutions of the target LAMP amplicons were allowed to hybridize with motors and the motion of the formed motor–DNA amplicon assemblies was tested in 5% H_2_O_2_ solution using the developed cellphone motion tracking system (Fig. 5c and Supplementary Movies 5 to 8). The results indicated that the presence of LAMP amplicons, even at a very low concentration of 3.394 ± 0.245 ng/µl, reduced the velocity of motors compared to control (no LAMP amplicons), considering signal-to-noise ratio = 3. We further tested the recovery of motor motion after releasing the target LAMP amplicons by incubating the formed LAMP DNA–motor assemblies at 90 °C for 30 s. The released amplicons were separated from the motors by centrifugation at 6000 × *g* for 5 min and tested in 5% H_2_O_2_ solution. There was a 75.52% recovery for the velocity of the tested motors (Supplementary Fig. 14). In addition, the response of the CALM system to non-target viruses was tested using different sexually transmitted RNA and DNA viruses commonly exist with HIV infection including hepatitis C virus (HCV), hepatitis B virus (HBV), herpes simplex virus type 1 (HSV-1), and human papillomavirus type 16 (HPV-16)^43^. The results of LAMP reaction confirmed that the DNA amplicons are only formed in the presence of the target HIV-1 and no visible amplification was observed with other tested viruses on agarose gel (Fig. 5d). Furthermore, the average velocity of motors in the presence of the amplification products of non-target viruses (i.e., LAMP reaction products generated with HCV, HBV, and HSV-1) was not significantly (*P* > 0.05, unpaired *t* test) different than control (no amplicons) samples and was at least three-folds higher than the average velocity of HIV-1 samples (Fig. 5e, f and Supplementary Fig. 15, 16). To further confirm the specificity of our system in the presence of the non-target amplicons or contamination, the motors were challenged with non-target LAMP amplicons generated from HPV-16 using specifically designed primers against envelop (E)-1 gene (Supplementary Table 2) and at amplification temperature of 60 °C for 30 min^44^. There was no significant change (*P* > 0.05, unpaired *t-* test) in the velocity of motors (*n* = 25) compared to control (no amplicons) in the presence of non-target amplicons confirming the high specificity of the developed CALM system for HIV-1 testing (Fig. 5e, f and Supplementary Figs. 15, 16).

### HIV-1 detection using the CALM system

**Fig. 6:**
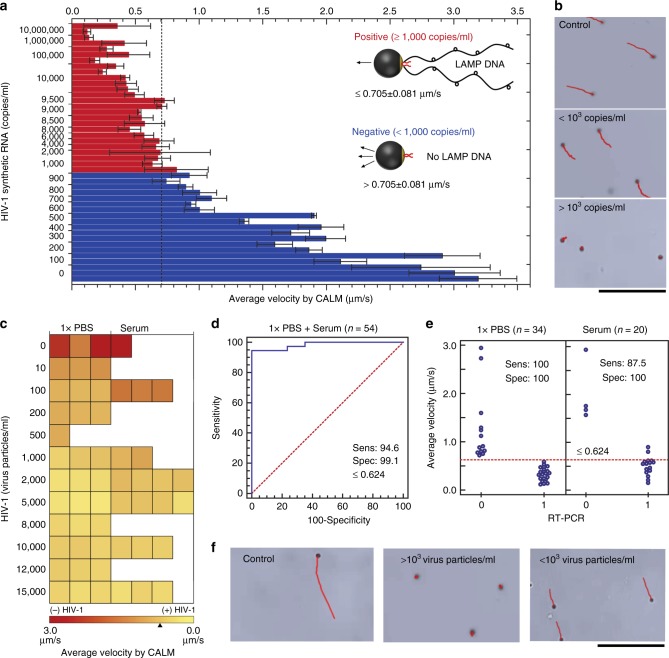
HIV-1 detection using the CALM system. Synthetic HIV-1 RNA standard detection: **a** bar graph shows the average velocity of motors recorded by the CALM system for phosphate-buffered saline (1× PBS, pH 7.4) samples (*n* = 45) spiked with different HIV-1 RNA concentrations. Vertical black dotted line indicates the average velocity at the threshold concentration of 1000 virus particles/ml; **b** representative digital images show the motion trajectories of motors in the absence of HIV-1 RNA (control) or the presence of HIV-1 RNA at concentrations above and below the threshold of 1000 copies/ml (scale bar = 100 μm). HIV-1 particles detection: **c** heatmap of the average motor velocity measured by the CALM system for different virus concentrations spiked in 1× PBS (*n* = 35) and serum (*n* = 20). The average velocity of HIV-1 positive samples (≥1000 virus particles/ml) is ≤0.704 ± 0.08 µm/s (yellow in color) and the average velocity of HIV-1 negative samples (<1000 virus particles/ml) is >0.704 ± 0.08 µm/s (red in color); **d** receiver-operating characteristics (ROC) curve analysis of 1× PBS (*n* = 35) and serum (*n* = 20) samples spiked with different HIV-1 concentrations showing the assay detection sensitivity (sens) and specificity (spec) compared to real-time polymerase chain reaction (RT-PCR). The threshold value for virus concentration applied here was 1000 particles/ml. Samples with virus concentrations above and below 1000 particles/ml were classified as positive and negative, respectively; **e** vertical scatterplot analysis of virus spiked samples (*n* = 54). The threshold value for average motor velocity applied here was 0.704 µm/s that corresponds to 1000 particles/ml. Samples with velocities above and below 0.704 µm/s were classified as 1 (positive) and 0 (negative), respectively; **f** representative digital images show the motion trajectories of motors in the absence of HIV-1 particle (control) and the presence of HIV-1 at concentrations above and below 1000 virus particles/ml (scale bar = 100 μm). The results are expressed as the average values of three independent experiments and error bars represent standard deviations

We evaluated the efficiency and reliability of the developed CALM system in HIV-1 detection using PBS (1× PBS, pH 7.4) and serum samples spiked with HIV-1 and patient plasma samples (Fig. 6 and Table 2). The developed system can qualitatively differentiate between samples with viral loads below (i.e., negative sample) and above (i.e., positive sample) a clinically relevant threshold value of 1000 copies/ml as recommended by the World Health Organization (WHO)^45^. To establish the motor velocity that corresponds to the threshold virus concentration of 1000 particles/ml, the system was first calibrated using 1× PBS samples (*n* = 48) spiked with different concentrations of stabilized synthetic HIV-1 RNA (0–10^7^ copies/ml). The prepared samples were amplified using LAMP and the generated amplicons were allowed to interact with motors for target capture and detection using the CALM system. The results demonstrated an average velocity of 0.705 ± 0.082 μm/s for samples with 1000 copies/ml and there was a significant difference (*P* < 0.0001, unpaired *t* test) between the average velocity of samples spiked with target RNA concentrations below and above the threshold value of 1000 copies/ml (Fig. 6a, b). Accordingly, the cellphone system was calibrated using this velocity value of 0.705 ± 0.082 μm/s to allow qualitative testing of PBS and serum samples spiked with virus particles (*n* = 54) (Fig. 6c–f, Supplementary Fig. 17 and Supplementary Movies 9–12). The qualitative results obtained by the CALM system compared to the standard quantitative real-time PCR (RT-PCR) technique are presented as heatmap in Fig. 6c. In addition, the receiver operating characteristic analysis (*n* = 54) showed that the CALM system has a sensitivity of 94.6% with a CI of 81.8–99.3% and a specificity of 99.1% with a CI of 80.5–100% at the threshold concentration of 1000 particles/ml. The area under the curve (AUC) was 0.984 with a binomial exact CI ranging from 0.905 to 1.00 and significance level *P* (area = 0.5) < 0.0001 (Fig. 6d). The vertical scatter plot analysis showed that the accuracy of the CALM system in correctly classifying PBS (*n* = 34) and serum (*n* = 20) samples spiked with HIV as positive and negative were 100% and 90%, respectively (Fig. 6e). The specificity and reliability of the developed CALM system was evaluated using serum samples spiked with HIV-1 and non-target viruses of HCV, HBV, and HSV-1. The results showed that the velocity of motors (*n* = 30) significantly (*P* < 0.01, unpaired *t* test) decreased in the presence of the HIV-1, while in the presence of non-target viruses the velocity of motors was not statistically different (*P* > 0.05, unpaired *t* test) than HIV-free control samples (Supplementary Fig. 18, 19). In addition, we evaluated the performance of the CALM system in identifying HIV-infected patient plasma samples compared to the results obtained by iSCA assay, which is a quantitative real-time PCR assay with single-copy sensitivity targeting a highly conserved region of integrase in the HIV-1 *pol* gene widely used in clinical diagnosis of HIV infection and ART monitoring (Table 2, Supplementary Fig. 20, 21 and Supplementary Movies 13, 14)^46^. We observed 100% accordance between the CALM system and iSCA assay in classifying patient plasma samples as positive (≥1000 virus particles/ml) and negative (<1000 virus particles/ml).

**Table 2:** CALM system evaluation using HIV-infected patient serum samples

Sample no.^a^	iSCA assay (copies/ml)^b^	CALM system (negative/positive)^c^
1	0	Negative (2.675 ± 0.424 μm/s)
2	0	Negative (2.079 ± 1.014 μm/s)
3	375	Negative (1.335 ± 0.144 μm/s)
4	540	Negative (0.913 ± 0.150 μm/s)
5	19,958	Positive (0.141 ± 0.014 μm/s)
6	136,366	Positive (0.219 ± 0.041 μm/s)

^a^Sample 5 was prepared by diluting sample 6 in 4

^b^iSCA assay is a quantitative real-time PCR assay with single-copy sensitivity targeting a highly conserved region of integrase in the HIV-1 *pol* gene

^c^Sample with velocity magnitude ≥0.705 ± 0.08 is positive (for viral load ≥1000 particles/ml) and  ± 0.08 is negative (for viral load  particles/ml), considering the average velocity of motors in samples spiked with synthetic HIV-1 RNA standard at the threshold value of 1000 copies/ml is 0.705 μm/s. The results are expressed as the average values of three independent experiments ± standard deviations

## Discussion

Early diagnosis of acute HIV infection is crucial since viral replication and shedding occur in this stage before detectable HIV antibodies appear^47^. Individuals with acute HIV infection who are unaware of their disease can contribute to HIV transmission. Most of the point-of care (POC) HIV diagnostics such as lateral flow assays, including dipsticks or enzyme immunoassays (ELISA), and OraQuick HIV test kit target the detection of antibodies against HIV and thus lack the capability to detect acute HIV infection even at high viral load of HIV^48,49^. In addition, the widespread implementation of ART has been highly effective in reducing mortality in HIV-infected individuals but the number of AIDS-related deaths is still >1.1 million every year. The recent treat-all policy of the WHO recommends earlier ART initiation regardless of CD4 count. Expanding access to ART in developing countries has cumulatively averted 5.5 million HIV-related deaths in low- and middle-income countries^50,51^. The updated guidelines will significantly increase the number of individuals eligible for ART^52^. Nucleic acid testing (NAT) for HIV viral load represents the most important method for the diagnosis of acute HIV infection and ART failure monitoring, but current NAT-based technologies cannot be implemented at the POC due to the need for specialized laboratory facilities and technicians. The limitations of current HIV viral load assays also represent barriers for the monitoring and detection of treatment failure in resource-limited settings. Thus there is an immediate need for an easy-to-use, portable, and inexpensive diagnostic tool for detecting acute HIV and early failure of ART. The presented cellphone-based motion sensing platform allows HIV detection by uniquely combining three technologies: (i) LAMP-based NA amplification, (ii) simple optical signal generation using micromotors (PS beads functionalized with AuNPs and PtNPs conjugated with antibodies), and (iii) cellphone-based optical sensing. Such system does not require bulky, expensive fluorescent-based optical components usually used in other commercially available NAT-based methods. This platform allows specific qualitative HIV detection with a threshold of 1000 virus particles/ml and thus can be implemented to detect acute infection and reduce the risk of virus transmission in the window period and to detect early treatment failure. We attribute the specificity and sensitivity of the developed system to the nature of LAMP technique used in nucleic acid amplification and the design of catalytic motors. We optimized LAMP amplification conditions (increasing target concentration and amplification reaction time) to amplify the target nucleic acid down to 1000 virus particles/ml using a set of four specific primers, which is directly contributing to the selectivity and specificity for the detection of HIV-1^43^^,^^44^. Engineering the surface of Pt-motors with 30-mer DNA capture probe that specifically recognize the target amplicon in a postamplification-capture step limits the binding of any non-specific amplicons and further improves the detection specificity and reliability. In addition, the CALM system takes the advantage of the highly efficient and rapid amplification of LAMP with a sensitivity limit down to single copy of the target. Together with the unique large-sized looped DNA amplicons characteristic to LAMP, the generated amplicons can effectively reduce the velocity of Pt-motors, allowing a rapid and sensitive detection of HIV-1 RNA^41,53^.

In our system, Pt-motors were prepared using PS beads with density of 1.1 g/cm^3^ to minimize the effect of gravity forces on motors facilitating their use as a motion sensor. Compared to bulk materials, NPs are known to possess higher surface-to-volume ratio and thus higher surface activity^54,55^. For this reason, PtNPs with average size <4 nm were used in the preparation of the motors to allow efficient catalytic activity^55,56^. In addition, the use of PtNPs allows better-controlled coverage ratio of the total surface of beads using thiol–metal chemistry compared to the commonly used metal sputtering technique that requires specific equipment and tedious control procedures over beads during the modification step. With ~4% surface coverage, the prepared Pt-motors were moving with an average velocity of 4.96 ± 0.281 μm/s in 10% H_2_O_2_ solution. Considering that our system relies on the payload effect of LAMP amplicon to change the velocity of motors, the control of the surface coverage of beads with PtNPs was critical to control the motor motion, allowing sensitive detection of the amplicon. It is worth mentioning that controlling the beads surface coverage with PtNPs helped to limit the accumulation of O_2_ bubbles generated during the catalytic-based motion of motors and allowed better imaging and motion tracking using the cellphone. The use of AuNPs to indirectly engineer motors with DNA capture probes allows efficient control on the number and position of the added DNA probes to the surface of Pt-motors. The number of DNA capture probe was optimized to be ~15 DNA molecules per motor particle to control the loaded number of the target amplicons and in turn the detection sensitivity of the developed system. Furthermore, this strategy of indirect modification of the motors with DNA allows spatial control over DNA probes to act as amplicon-binding spot that induces the accumulation of amplicons in one side of the motors to allow more directional and uniform motion of the motors. This is critical to allow easy motion tracking by cellphone and enhanced and reliable sensing performance of the developed CALM system.

The presented work has demonstrated the development of a cellphone-based technology for HIV-1 RNA detection based on monitoring the change in the velocity of catalytic micromotors in H_2_O_2_ solution when they interact with DNA amplicons. LAMP technique allows target amplification under isothermal condition and the used micromotors are simple in design and can easily be prepared using commercially available PS beads and metal NPs. The material cost of the hardware cellphone accessory, microchip, and reagents per test is <$5 (Supplementary Table 3). We showed that the developed system can rapidly (<1h) and qualitatively classify HIV-infected samples based on a threshold of 1000 copies/ml with 90–100% accuracy as compared to RT-PCR. In addition, this system is affordable, sensitive, specific, rapid, and user-friendly, which are the major components of ASSURED criteria. Thus it has significant advantages in addressing an unmet clinical need in HIV detection and treatment monitoring and can potentially prevent unintended infection transmission. However, the presented system is a platform technology and will need additional modifications to allow sample processing and amplification. Further testing using the standard laboratory-based methods will be needed to confirm positive results generated by the presented cellphone system before initiating any treatment. The developed platform can be used in molecular assays that require simple, low-cost, and rapid testing of viruses. The proposed system has a great potential for broad applications in infectious diseases control and management.

## Methods

### HIV-1 propagation and nucleic acid isolation

HIV-infected peripheral blood monoclonal cells (PBMCs) were first isolated from patient blood samples using Ficoll-Hypaque density gradient cell centrifugation. PBMCs were then stimulated by phytohemagglutinin and co-cultured with irradiated PBMCs at 37 °C and 5% of CO_2_. The virus titer in the co-culture supernatant was tested using HIV-1 p24 antigen ELISA (PerkinElmer Life Science, Inc., NEK050b). The co-culture process was continued till the concentration of p24 became 20 ng/ml. The cell culture supernatant was collected, and the virus concentration was tested using Roche-COBAS AmpliPrep TaqMan HIV-1 v2.0 system at Brigham and Women’s Hospital (BWH). For sample testing with the cellphone system, HIV-1 RNA was isolated from each sample using the AllPrep DNA/RNA Mini Kit (Qiagen, CA, USA) following the manufacturer's protocol.

### Microchip fabrication

A single-channel microchip consists of three layers: (1) PMMA (3.175 mm; McMaster-Carr Inc., 8560K239) that contains the inlets and outlets of microchannels, (2) DSA sheet (80 μm; 3M Inc., 82603) that includes the microfluidic channel, and (3) glass slide (25 ×75 mm^2^; Globe Scientific Inc., 1358A). The microchip design was initially prepared using the vector graphics editor CorelDraw X7 software. Then the DSA and PMMA were machined using the VLS 2.30 CO_2_ Laser cutter (Universal Laser systems AZ). The DSA was used to assemble PMMA and glass slide and the prepared chips were cleaned and tested for leakage using de-ionized water.

### Pt-motor preparation and characterization

Platinum micromotors were prepared of spherical 6-μm PS beads coated with PtNPs and AuNPs and modified with DNA capture probe that recognizes HIV-1 LAMP amplicons. The detailed protocol includes three main steps: (1) PtNPs and AuNPs synthesis, (2) DNA conjugation to AuNPs, and (3) PS beads surface activation and sequential coating with NPs. The synthesis of PtNPs and AuNPs was performed following the common protocol of metal salt reduction with sodium borohydride^57–59^. For PtNPs synthesis, 100 ml of ultrapure water was heated in a 250-ml Erlenmeyer flask and brought to boiling and 7.2 ml of a 0.2% chloroplatinic acid hexahydrate solution was added and mixed by magnetic stirring. Then 2.2 ml of 1% sodium citrate freshly prepared in 0.05% citric acid was injected in the flask and the solution was mixed for 1 min. In all, 1.1 ml of 0.08% sodium borohydrate solution freshly prepared in 1% sodium citrate–0.05% citric acid solution was added while boiling and the reaction continued till the formation of the PtNPs. For AuNP synthesis, a seed solution of ~15 nm-AuNPs was first prepared by adding 900 μl of 1% sodium citrate trihydrate solution to 300 μl of 1% HAuCl_4_ diluted in 30 ml of H_2_O. The growth reaction of AuNPs was then initiated by adding 391 μl NP seed solution to 100 μl of 1% (W/V) HAuCl_4_ diluted in 9.5 ml of H_2_O under rapid stirring at room temperature followed by the addition of 22 μl of 1% sodium citrate solution and 100 μl of 0.03 M hydroquinone. The reduction is completed within 10 min. One milliliter of the synthesized AuNPs was mixed with freshly reduced thiolated-DNA probe deigned against HIV-1 *gag* gene (50 μM) and the mixture was incubated at room temperature for 12 h. The solution was then brought to 0.1 M NaCl and allowed to stand for 40 h and washed twice by centrifugation at 12,000 × *g* for 30 min using 10 mM phosphate buffer (pH 7.2)^60^. To prepare thiolated beads, 0.14 pM amine-functionalized PS beads (Spherotech, Inc., AP-60–10) were mixed with 1.6 mM SPDP crosslinker (Thermo Fisher Scientific Inc., 21857) in phosphate buffer (pH 7.2) and incubated for 3 h at room temperature. Then the thiolated beads were first coupled with the prepared DNA–AuNP conjugates using the well-known thiol–gold chemistry followed by adding excess of PtNPs to coat the remaining surface of beads. The prepared Pt-motors were characterized using TEM, UV-vis spectroscopy, FT-IR, Zeta potential (*ζ*), DLS, and ICP-MS^59^.

### LAMP reaction

RT-LAMP amplification of the target HIV-1 RNA was performed using a set of four specific primers (Table 1). The reaction was performed as follows: a mixture of the 4 sets of DNA primers (50 µM) was first prepared by mixing 0.8 µl of FIP, 0.8 µl of BIP, 0.1 µl of F3, and 0.1 µl of B3 and then added to the reaction mixture prepared of 2.5 µl isothermal amplification buffer (New England Biolabs Inc., BO537S), 1.5 µl MgSO_4_ (100 mM), 1.4 µl dNTP (25 mM), and 2.5 µl Betaine (5 M). Then 2–4 µl of the target and non-target RNA was added followed by adding of 6 unit of AMV reverse transcription enzyme (New England Biolabs Inc., M0277L) and 8-unit Bst. 2.0 DNA Polymerase (New England Biolabs Inc., M0537L). The reaction volume was brought to 25 µl by UltraPure™ DNase/RNase-Free Distilled Water (Thermo Fisher Scientific Inc., 10977023) and mixed thoroughly before incubation for 40–50 min at 65 °C and termination at 85 °C for 5 min^42^.

### LAMP amplicon capture and motion assay

Our assay relies on reducing the motion of the Pt-motors when specifically coupled with the large-sized LAMP amplicons. The prepared LAMP amplicons are hybridized with Pt-motor at 80 °C for 2 min and cooled to 4 °C^61^. Then 10 µl of the formed assemblies were mixed with H_2_O_2_ solution and loaded on the microchip. The reduction of the beads motion in the presence of HIV-1 LAMP amplicons was tested using either the developed cellphone system or the bright-field light microscopy (Carl Zeiss AG Axio Observer D1) using Snagit v11.4.3 (Build 280) video recording software. The recorded videos were analyzed using ImageJ and MtrackJ plug-in to manually calculate the velocities of beads in the tested sample.

### Motor tracking cellphone system

The cellphone attachment was designed using the Solidworks 2015 software and 3D printed using Ultimaker Extended II 3D printer and Ultimaker PLA as printing material. The cellphone attachment was designed to record the videos using the cellphone rear camera of a Moto X smartphone (Motorola, XT1575). The optical cellphone attachment has an LED, electronics, and switches and two acrylic lenses extracted from TS-H492 discarded optical drives with focal lengths of 4 and 27 mm and numerical apertures of 0.43 and 0.16^62^. The cellphone application was designed using Android Studio to record a video of the sample for 30 s at 30 frames/s. The detection algorithm identifies the motors and tracks its motion to calculate its average velocities. The presence of the target virus is then determined based on the change in bead motion in the tested sample.

### Evaluation of the CALM system in HIV-1 detection

We evaluated the effect of the presence of different concentrations of HIV-1 LAMP amplicons prepared by diluting the final amplification product in PB (pH 7.2) into the following percentages 100, 50, 10, 1, 0.5, 0.1, 0.01, and 0.0%. The total DNA concentration in each dilution was first measured using a NanoDrop One-C spectrophotometer (Thermo Fisher Scientific Inc.) and 10 µl of each concentration was mixed with Pt-motors and tested using the CALM system. We evaluated the performance of the CALM system using HIV-1 and non-target viruses, including HCV, HBV, HSV-1, and HPV-16. The cellphone system was calibrated with PBS samples spiked with synthetic HIV-1 RNA standard (0–1 × 10^7^ copies/ml) purchased from ATCC (VR-3245SD) and then compared to the standard RT-PCR using 1× PBS (pH 7.4) and serum samples spiked with HIV-1 particles at concentrations between 0 and 1.5 × 10^4^ virus particles/ml. In addition, the developed CALM system was tested using HIV-infected patient serum samples (*n* = 4) and fresh whole blood from HIV-negative subjects (*n* = 2) purchased from Research Blood Components Inc. HIV-1 plasma samples were prepared from whole blood obtained from patients enrolled in the HIV-1 Eradication and Latency (HEAL) Cohort and ART treated and followed up at BWH and Massachusetts General Hospital. This study was approved by the Partners Human Research Committee. Participants of the HEAL cohort represented a convenient sample of participants meeting the HEAL inclusion criteria. Samples obtained were based on participant flow and no other sample selection criterion was in place for the study. All patients (HIV positive and negative) provided informed consent for blood samples to be collected.

### Statistical analyses

Statistical analyses were performed using OriginPro 2015 (OriginLab Corporation, Northampton, USA), GraphPad Prism software version 5.01 (GraphPad Software, Inc. La Jolla, CA, USA), and MedCalc 14.8.1 (MedCalc Software bvba, Ostend, Belgium). Correlation between the motion tracking cellphone application and the bright-field microscopy was performed using linear regression analysis, and paired *t* test analysis was used to compare the motor motion analyzed by both techniques. All data for system performance were analyzed using unpaired *t* test. Differences between groups were considered significant when *P* values were not >0.05, and levels of significance were assigned as *P ≤ 0.05, **P ≤ 0.01, ***P ≤ 0.001, and ****P < 0.0001. Each data point represents the average of a total of three independent measurements.

### Code availability

The computer code used for motion tracking in the developed cellphone system reported in this study is available upon request from the authors.

## Electronic supplementary material

Supplementary Information

Supplementary Movie 1

Supplementary Movie 2

Supplementary Movie 3

Supplementary Movie 4

Supplementary Movie 5

Supplementary Movie 6

Supplementary Movie 7

Supplementary Movie 8

Supplementary Movie 9

Supplementary Movie 10

Supplementary Movie 11

Supplementary Movie 12

Supplementary Movie 13

Supplementary Movie 14

Description of Additional Supplementary Information

## Data Availability

All relevant data are included within the article and Supplementary Information files and available upon reasonable request from the authors.
