# Supplementary material for: DNA engineered micromotors powered by metal nanoparticles for motion based cellphone diagnostics
Source: Nat Commun. 2018 Oct 16;9:4282. doi: 10.1038/s41467-018-06727-8 (PMC6191441; doi:10.1038/s41467-018-06727-8)
Supplement: Supplementary file 1 — Supplementary Information [file 41467_2018_6727_MOESM1_ESM.pdf]

Supplementary Information

DNA engineered micromotors powered by metal nanoparticles for motion based cellphone diagnostics

Draz et al.

1. Supplementary methods

UV-Vis Spectroscopy. Absorption spectra were measured on Beckman Coulter DU 800 UV-Vis spectrophotometer. The samples were loaded in 10 mm path length quartz cuvettes and scanned at room temperature.

Transmission Electron Microscopy (TEM). Transmission electron microscopy images were obtained using a JEOL 2100 TEM microscope at an acceleration voltage of 300 kV. The specimens were prepared by dropping 2 μl of the sample onto ultrathin Formvar-coated 200-mesh copper grids and then dried in air. The mean diameter and size distribution histogram of particles were obtained by averaging more than 100 particles from the TEM images using ImageJ software.

Zeta Potential and Dynamic Light Scattering. Zeta potential and dynamic light scattering (DLS) experiments were performed using Malvern zetasizer (Malvern Instruments, Malvern, UK). The samples were initially filtered through a 0.22 μm filter membrane and allowed to settle overnight for DLS measurements. Ultrapure water (>18 MΩ) from a Pure Lab Ultra water system (ELGA, Ltd) was used as diluent and 3 measurements were recorded for each sample at room temperature. The average values were calculated from the reported results for three different batches.

Fourier transform-infrared (FT-IR) spectroscopy. FTIR spectra in the region of 2000–500 cm^{-1} were collected in absorbance mode with a FTS 135 BIO-RAD FT-IR spectrometer.

Inductively coupled plasma mass spectrometry (ICP-MS). We performed ICP-MS analysis to confirm and evaluate Pt and Au metal concentration in motor structure. Standard solutions were prepared by diluting a multi-element standard (1000 mg/L in 1 M

HNO_3). Nitric acid (65%), hydrochloric acid (37%), perchloric acid (70%), and hydrogen peroxide (30%) of Suprapur®grade were used to mineralize the Pt and Au metal from the motors. These samples were then digested and purified using size-exclusion column of a HPLC system. The eluted fractions from the HPLC system were detected by ICP-MS (SCIEX ELAN 5000, Perkin Elmer) and the concentrations of Pt and Au were identified using the standards for platinum and gold.

Silver staining technique.

Silver staining technique was used to confirm the presence of DNA-AuNP conjugates on the surface of beads using HQ Silver Enhancement Kit (Nanoprobes Inc., NY) following the manufacturer's protocol. Briefly, equal volumes of the initiator (A), moderator (B), and the activator (C) were mixed thoroughly in a dark environment and 50 µl of the prepared solution was immediately mixed with 50 µl of DNA AuNP-beads sample and control samples of beads (no AuNPs were added) in a microtitre plate. The plates were then incubated in a dark environment to allow staining reaction and color development for 10 min. The wells were then imaged, and its intensity was measured using BioTek plate reader.

Fluorescence imaging of LMAP amplicon on glass slide for evaluating LAMP amplicons capture using Pt-motors.

LAMP amplicons that are captured by motors, isolated by centrifugation, and released by thermal dehybridization were stained with GelRed stain solution (1 µl of stain in 50 ml DI water) and compared to LAMP amplicon solutions with known concentrations to determine the capture efficiency of the motors. 10 µl of stain solution was mixed with 10 µl of

amplicon solution. Then 10 μl of the resultant mixture were dropcasted on the surface of a glass slide and imaged using G-Box gel documentation system.

Quantification of the number of DNA capture-probes per AuNPs. The concentration of DNA probes conjugated to AuNPs was estimated using UV-vis spectroscopy. A standard curve was prepared from aliquots containing known concentrations of DNA probes and absorbance values were measured at 260 nm with a BioTek microplate reader. To calculate the number of DNA probes, the absorbance value of AuNPs was subtracted from the absorbance measured for DNA-AuNP conjugates of equal particle concentration to correct background and then the concentration of DNA capture probe in the conjugates solution was estimated from a linear curve prepared from known DNA concentrations. The number of DNA probes on each AuNP was calculated by dividing the concentration of DNA to the concentration of the AuNPs.

Agarose gel electrophoresis. The gel was prepared prior to each LAMP reaction by mixing 1.4 g of Agarose gel with 120 ml of Tris-Acetate-EDTA buffer (Sigma-Aldrich) TAE 1X buffer and 3 μl Gel red. The prepared gel was then stored at room temperature until it got solidified. After the electrophoresis tray was filled with 1% TAE buffer, gel sank inside the buffer and DNA sample mixed with loading dye was loaded. The gel was run for 40-60 min at 120V.

Image analysis with imageJ software. Particle tracking in videos recorded by a bright-field light microscope was performed through converting the videos into different frames using Virtual Hub 3.4 software. Then, these images were analyzed using a MTrackJ plugin for ImageJ. Briefly, image sequence of the video was imported to ImageJ and the motors

were tracked manually in each frame using MtrackJ plugin to calculate the displacement and the mean speed for each motor.

2. Supplementary figures

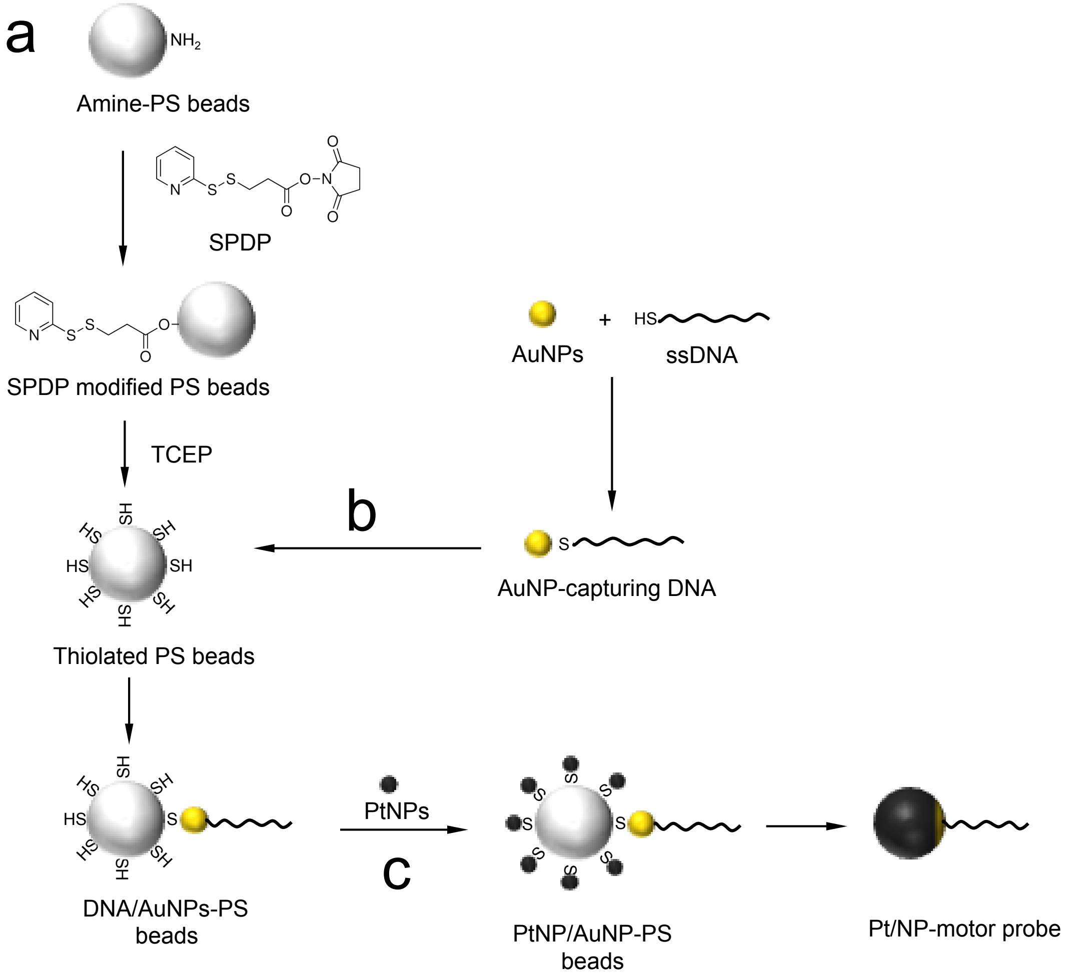

Supplementary Figure 1. Motor preparation. (a) Activation of the surface of polystyrene (PS) beads: 6-μm PS beads were activated with N-Succinimidyl 3-[2-pyridyldithio]-propionate (SPDP) crosslinker to prepare thiolated PS beads. (b) Addition of AuNPs-capturing DNA to the surface of thiolated PS. The molar ratio between AuNPs and PS was controlled to allow the preparation of PS carrying single Au-nanoprobe. (c) Addition of PtNPs: the remaining surface of the thiolated beads is covered with PtNPs. The coupling of both AuNPs and PtNPs to the surface of thiolated beads relies on the well-known thiol metal non-covalent binding.

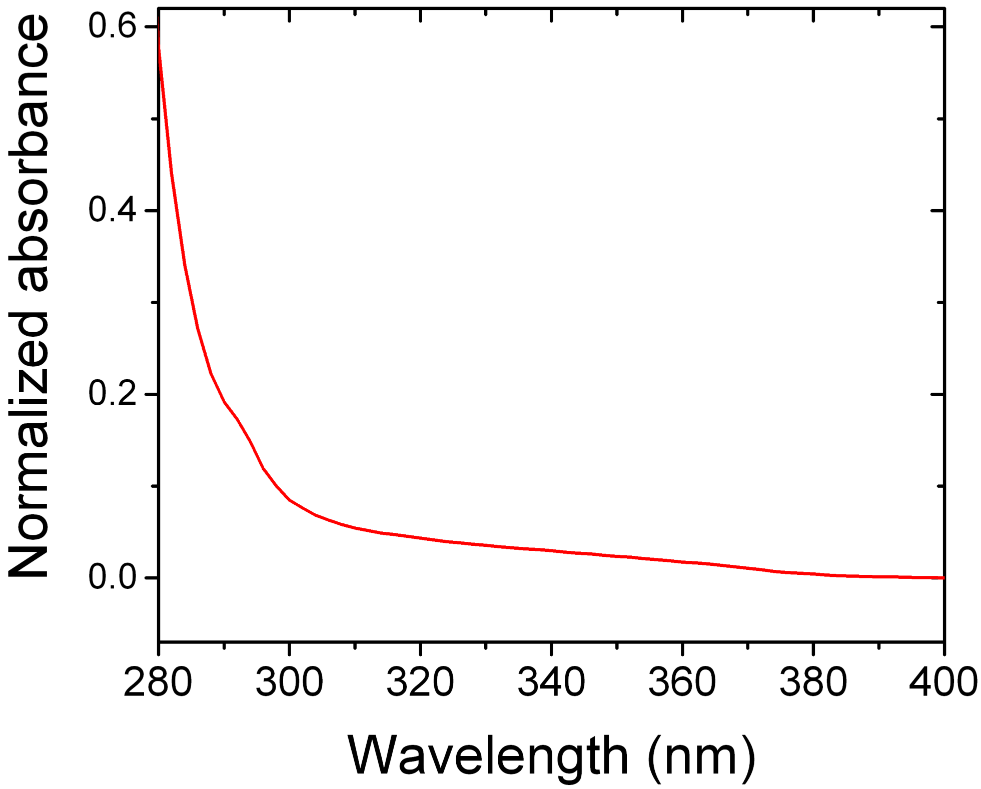

Supplementary Figure 2. UV-vis absorbance spectrum of PtNPs. The result show a characteristic band around 289 nm.

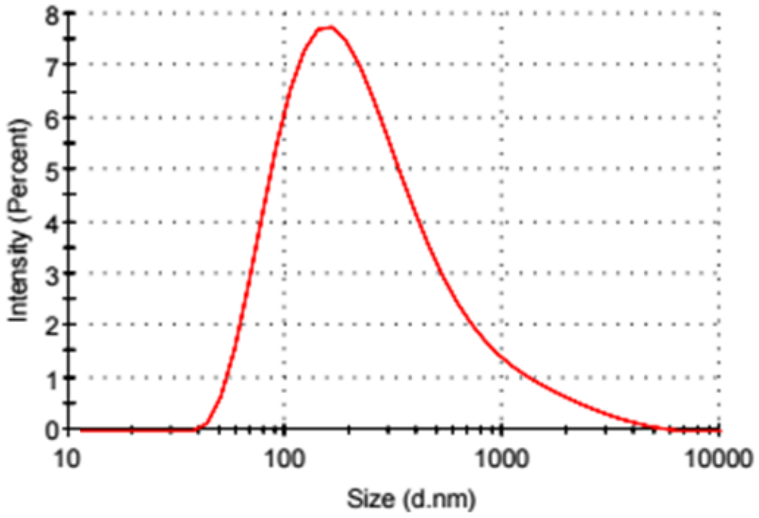

Supplementary Figure 3. Dynamic light scattering analysis of AuNPs. The results confirm the stability of the synthesized nanoparticles.

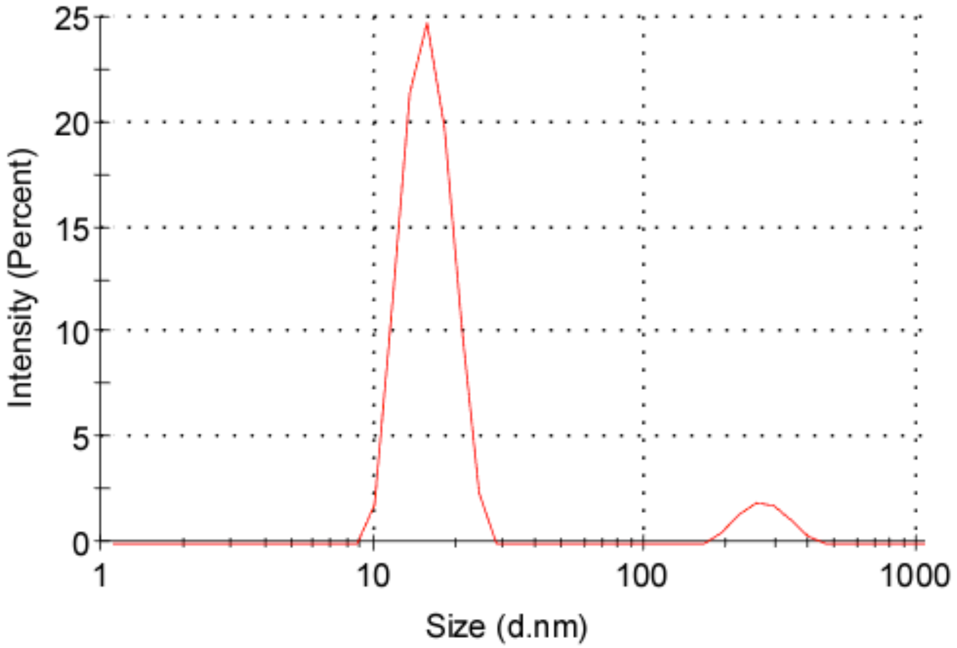

Supplementary Figure 4. Dynamic light scattering of PtNPs. The results confirm the stability of the synthesized nanoparticles.

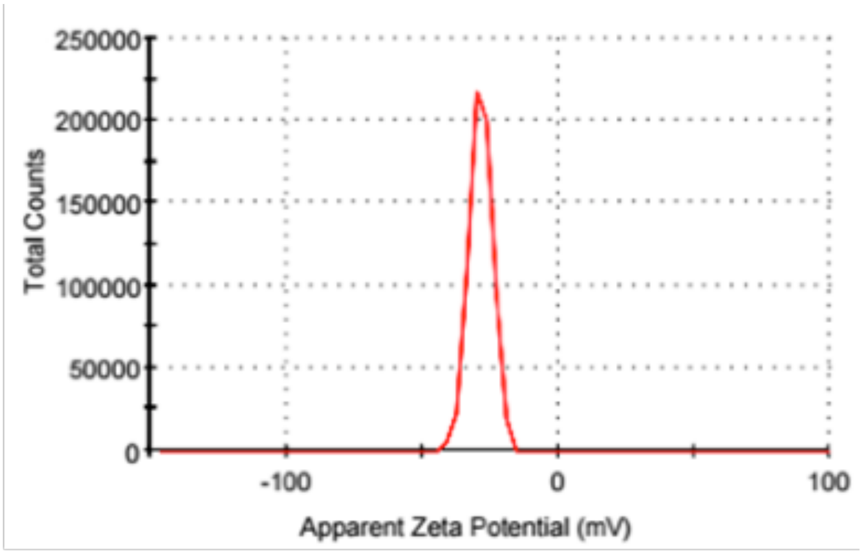

Supplementary Figure 5. Zeta potential analysis of the prepared AuNPs.

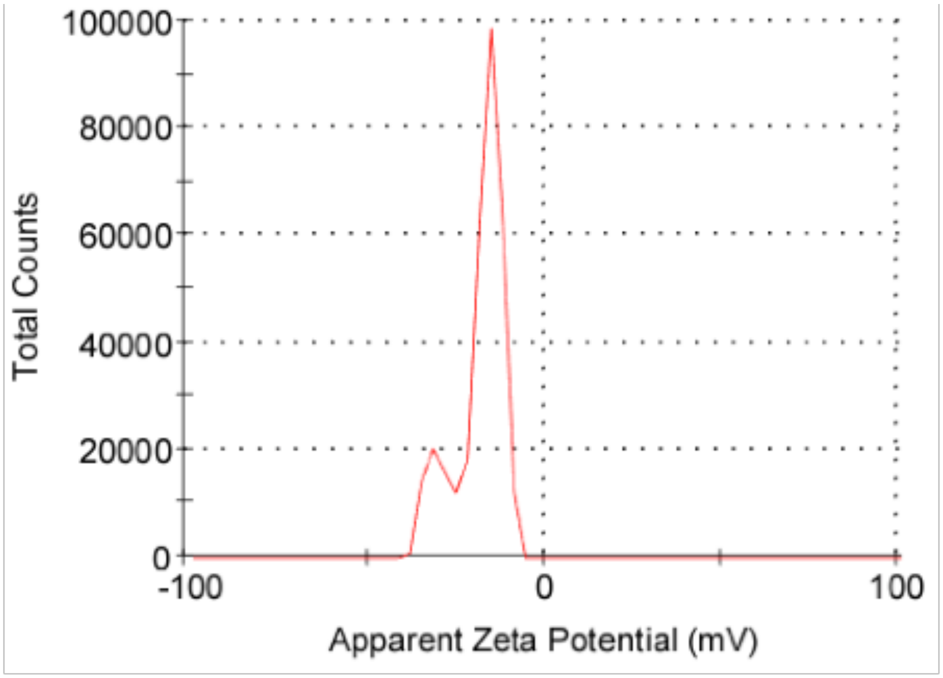

Supplementary Figure 6. Zeta potential analysis of the prepared PtNPs.

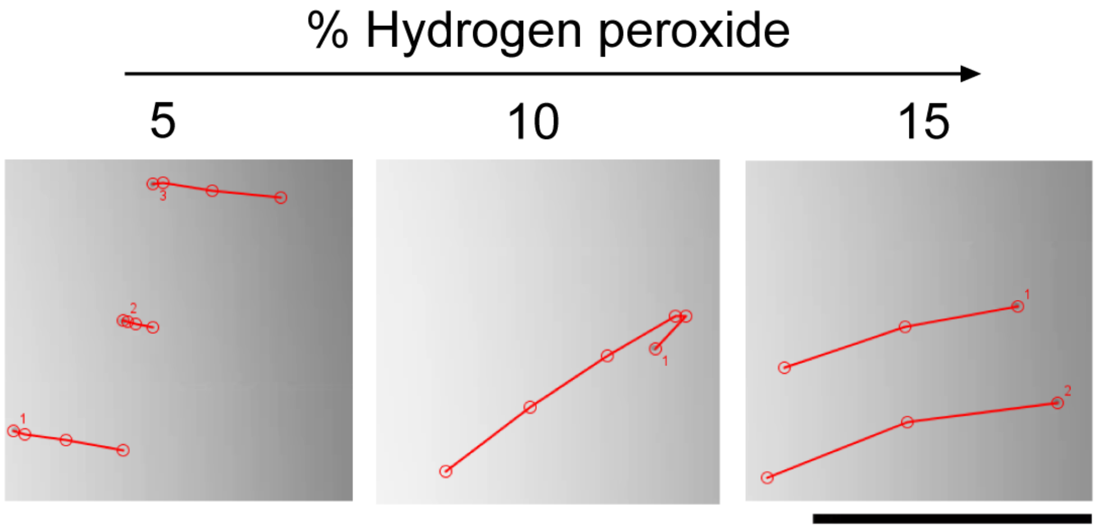

Supplementary Figure 7. The motion trajectory of Pt-motors in different H_2O_2 concentrations. The scale bar is 100 μm.

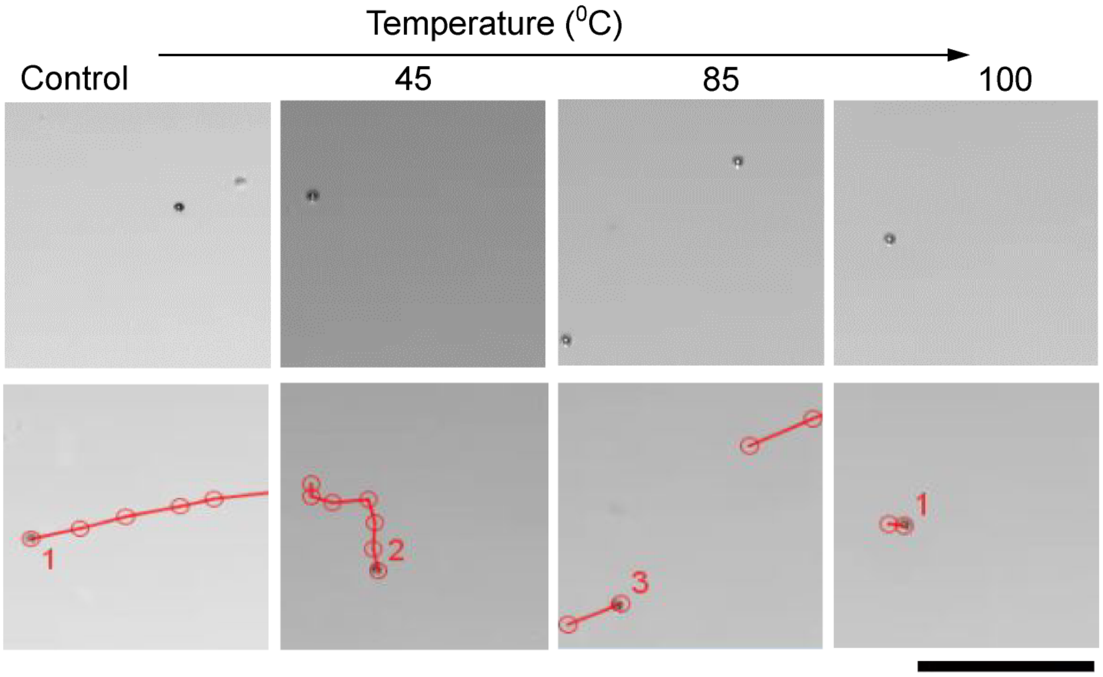

Supplementary Figure 8. Evaluation of the effect of temperature on the motion of Pt-motors. Motors are incubated at different tempratures for 10 min and then mixed in 5% H_2O_2 . The scale bar is 100 μm.

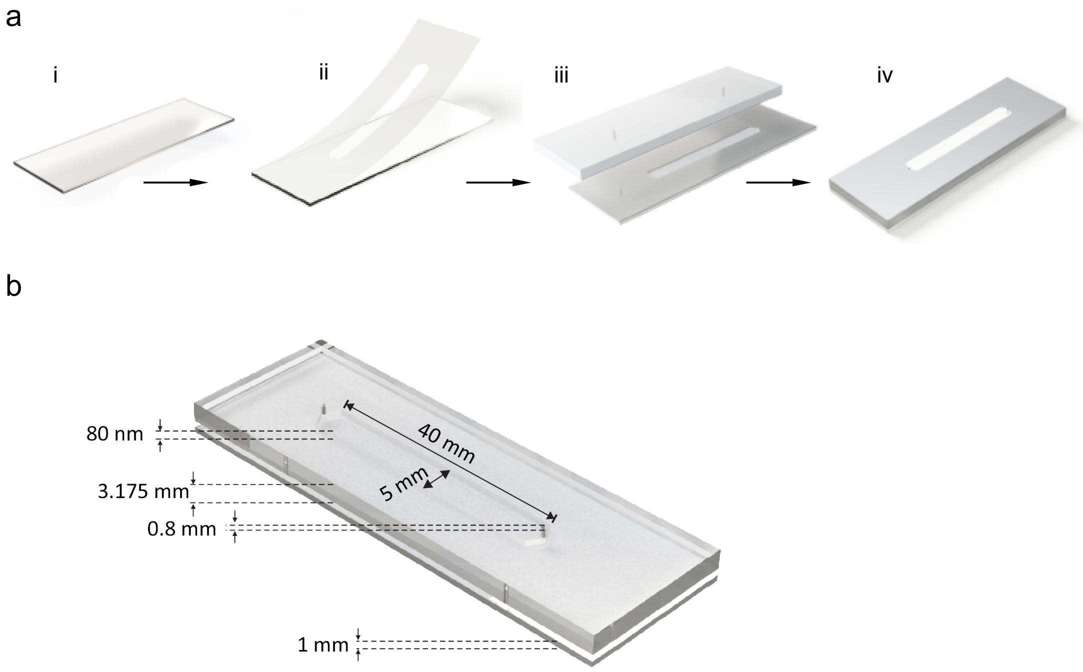

Supplementary Figure 9. Microchip fabrication and detailed design. (a) Microchip fabrication protocol: (i) glass slide cleaning with 75% ethanol and DI water; (ii) the addition of double-sided adhesive (DSA) layer on the cleaned glass slide; (iii) addition of poly(methyl methacrylate) (PMMA) layer on DSA-glass substrates; (iv) microchip testing for leakage. (b) Detailed design and dimensions of the microchip used in this study.

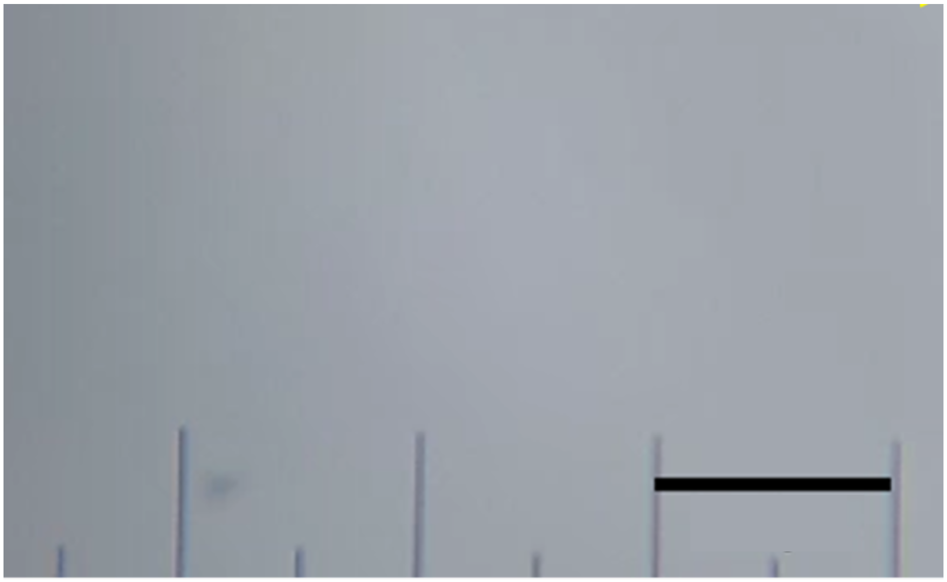

Supplementary Figure 10. Micrometer scale used for cellphone system calibration. The scale bar is 100 μm.

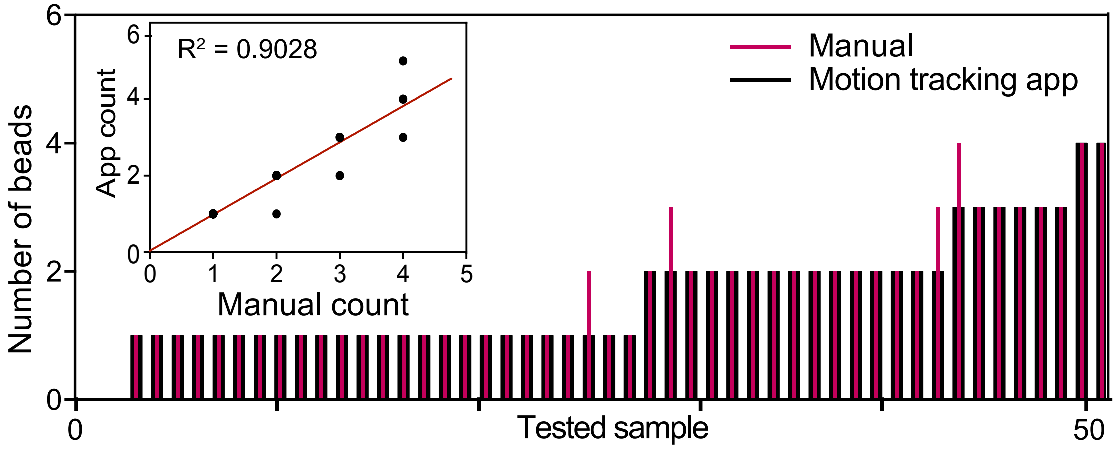

Supplementary Figure 11. Evaluation of the motion tracking smartphone application in counting Pt-motors. The results of motor counting using the cellphone motion tracking application is compared to the counts generated from bright-field light microscopy of the same motor samples (n = 50).

4261 agtagaatct atgaataaag aattaaagaa aattataggc caggtaagag atcaggctga
4321 acatcttaag acagcagtac aaatggcagt attcatccac aattttaaaa gaaaaggggg
4381 gattgggggg tacagtgcag gggaaagaat agtagacata atagcaacag acatacaaac
4441 taaagaatta caaaaacaaa ttacaaaaat tcaaaatttt cgggtttatt acagggacag
4501 cagagatcca ctttggaaag gaccagcaaa gctcctctgg aaaggtgaag gggcagtagt

Supplementary Figure 12. LAMP target sequence of human immunodeficiency virus 1 (HIV-1).

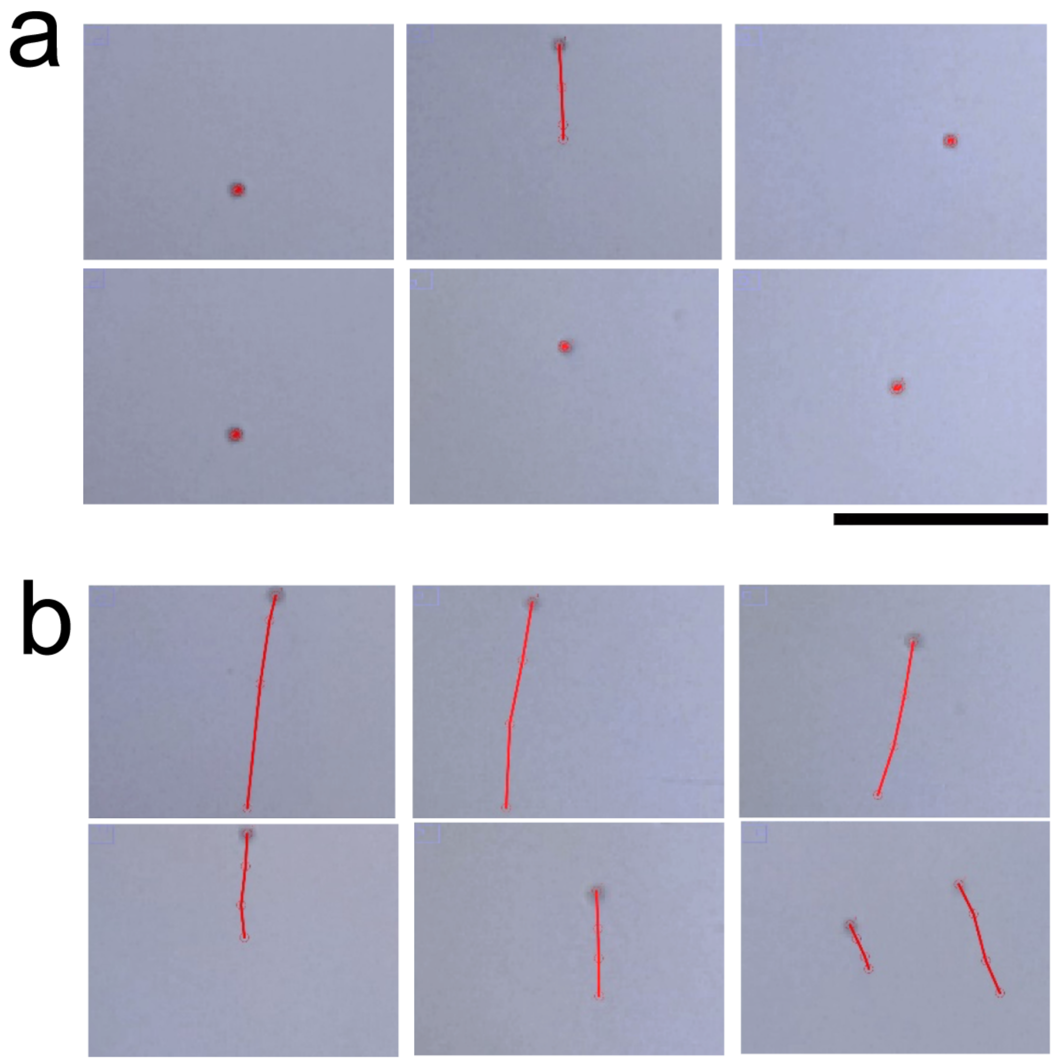

Supplementary Figure 13. Motors motion in the presence of target LAMP amplicons. Motion of Pt-motors in the presence (a) and absence (b) of the target human immunodeficiency virus 1 (HIV-1) LAMP amplicons. The scale bar is 100 µm.

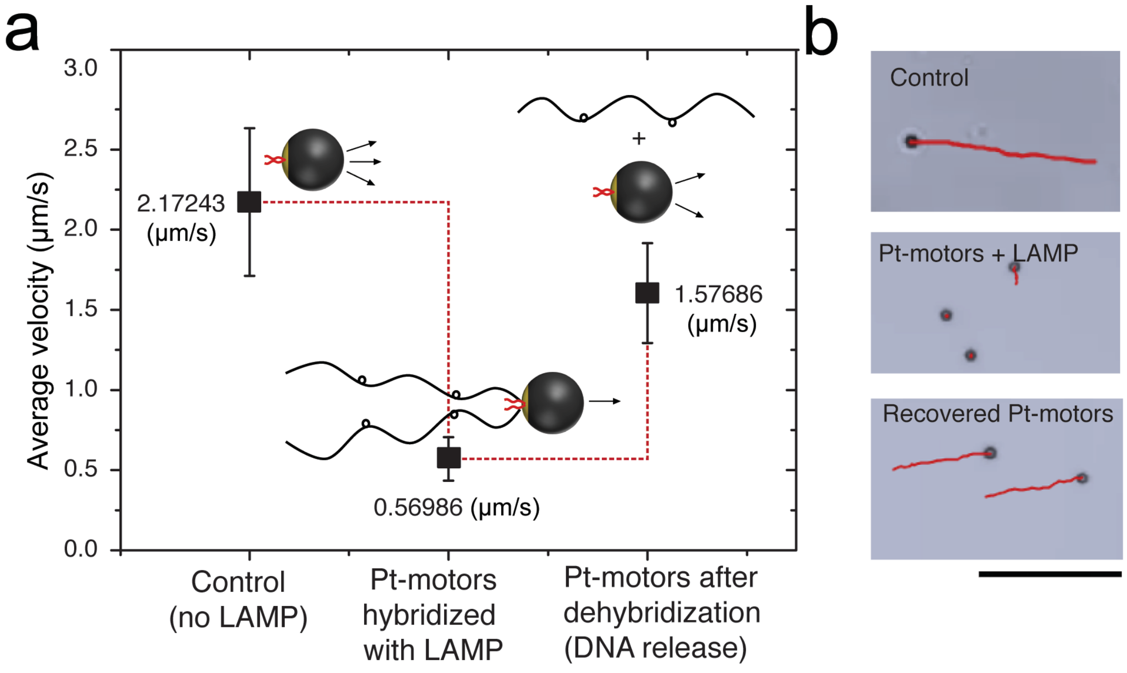

Supplementary Figure 14. Motion recovery of Pt-motors. (a) Horizontal step analysis shows average velocity of Pt-motors before assembly with the target HIV-1 LAMP amplicons, after assembly with LAMP amplicons (10^4 virus particles/ml was used as target), and after disassembly and release of LAMP amplicons by dehybridization at 90 ^{0}C for 30 s. (b) Representative digital images show the motion trajectories of motors at different steps. The scale bar is 100 µm.

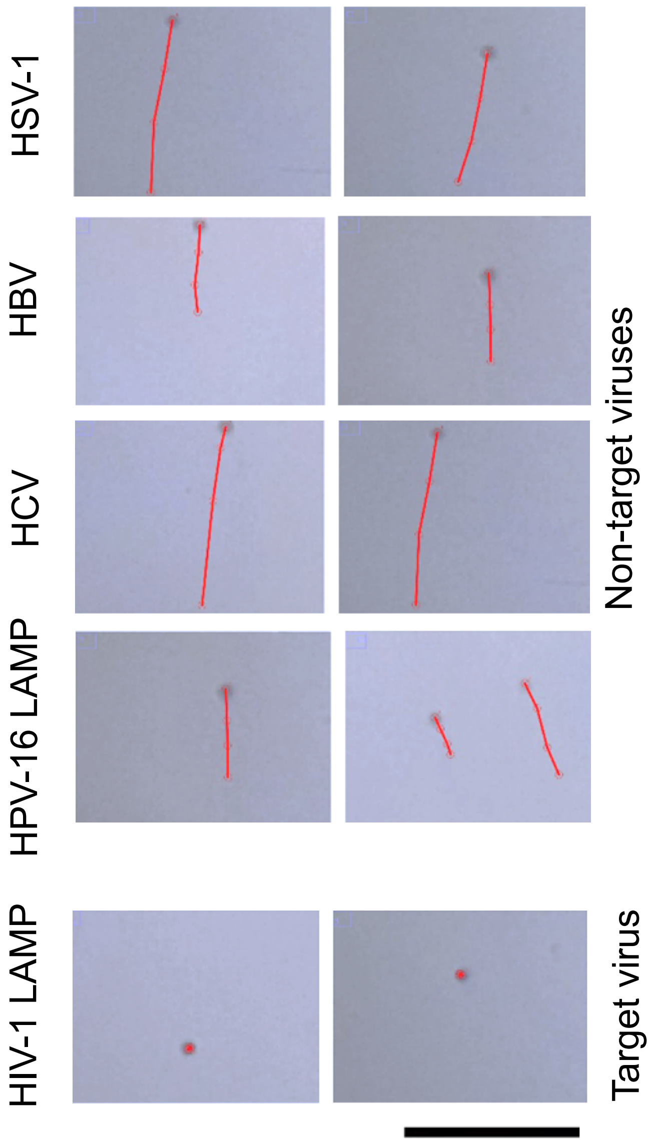

Supplementary Figure 15. Motors motion in the presence of non-target LAMP amplicons. Motion of Pt-motors in the presence of LAMP amplification products generated from target human immunodeficiency virus 1 (HIV-1) and the non-target viruses of hepatitis C virus (HCV), hepatitis B virus (HBV), human simplex virus 1 (HSV-1) and human papillomavirus 16 (HPV-16). The scale bar is 100 μm.

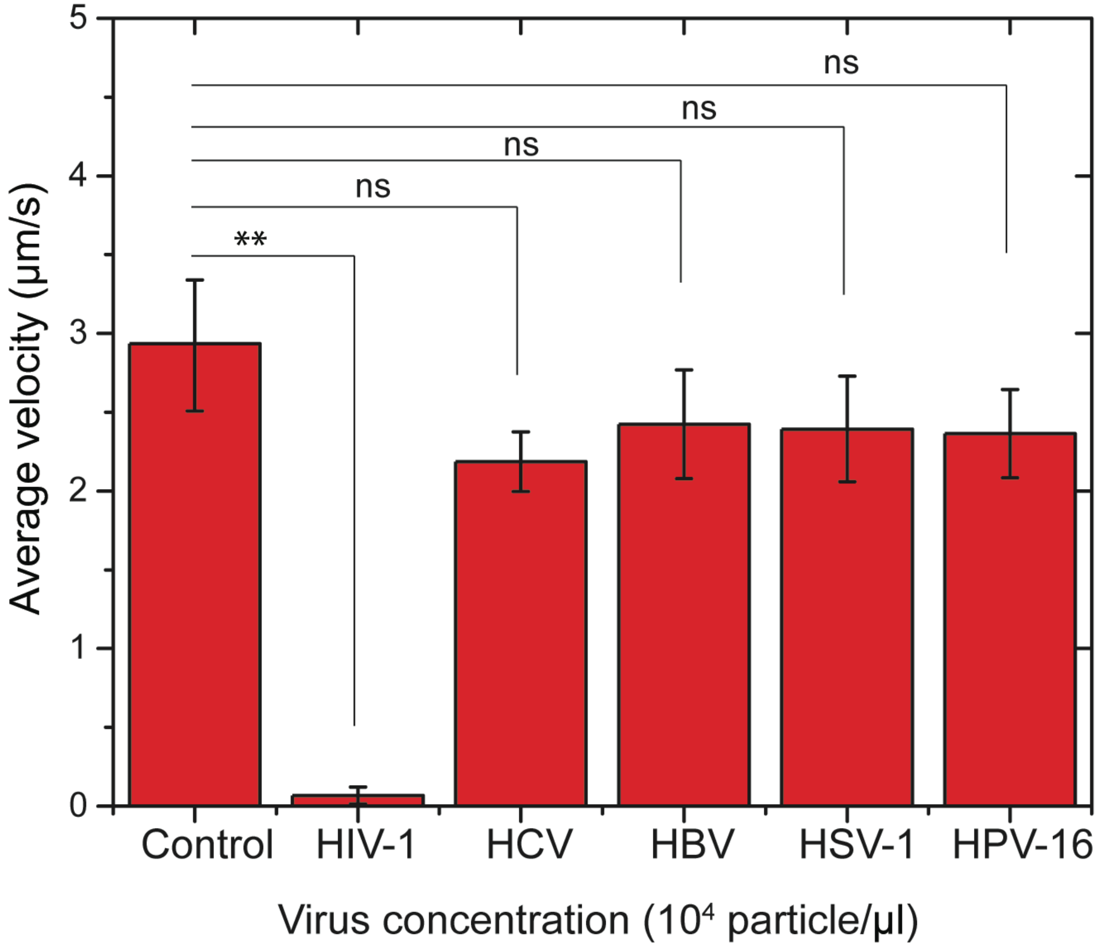

Supplementary Figure 16. Specificity testing of the CALM system virus spiked samples. 1x phosphate buffered saline (PBS) samples spiked with the target human immunodeficiency virus 1 (HIV-1) and the non-target viruses of hepatitis C virus (HCV), hepatitis B virus (HBV), and human simplex virus 1 (HSV-1), and the LAMP amplicon of human papillomavirus 16 (HPV-16) and control PBS samples (no virus) are used to test the detection specificity of the developed CALM system. The results are expressed as the average values of 3 independent experiments. Error bars represent standard deviations. $^{**}P < 0.01$; ns, nonsignificant versus the corresponding control group (no HIV-1), calculated using the unpaired t-test.

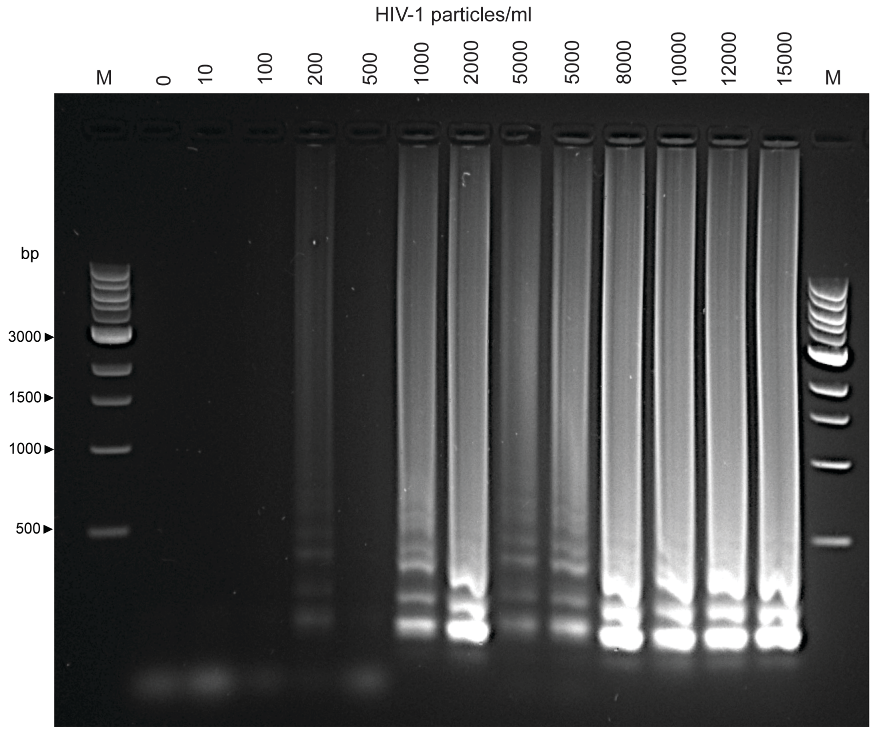

Supplementary Figure 17. Representative agarose gel electrophoresis image of LAMP amplification products of HIV-spiked samples. Phosphate buffered saline samples were spiked with different concentrations of HIV-1 particles (0- 15000 particles/ml) and used as target (4 µl per reaction) for LAMP amplification for 45 min at 65^{0}C.

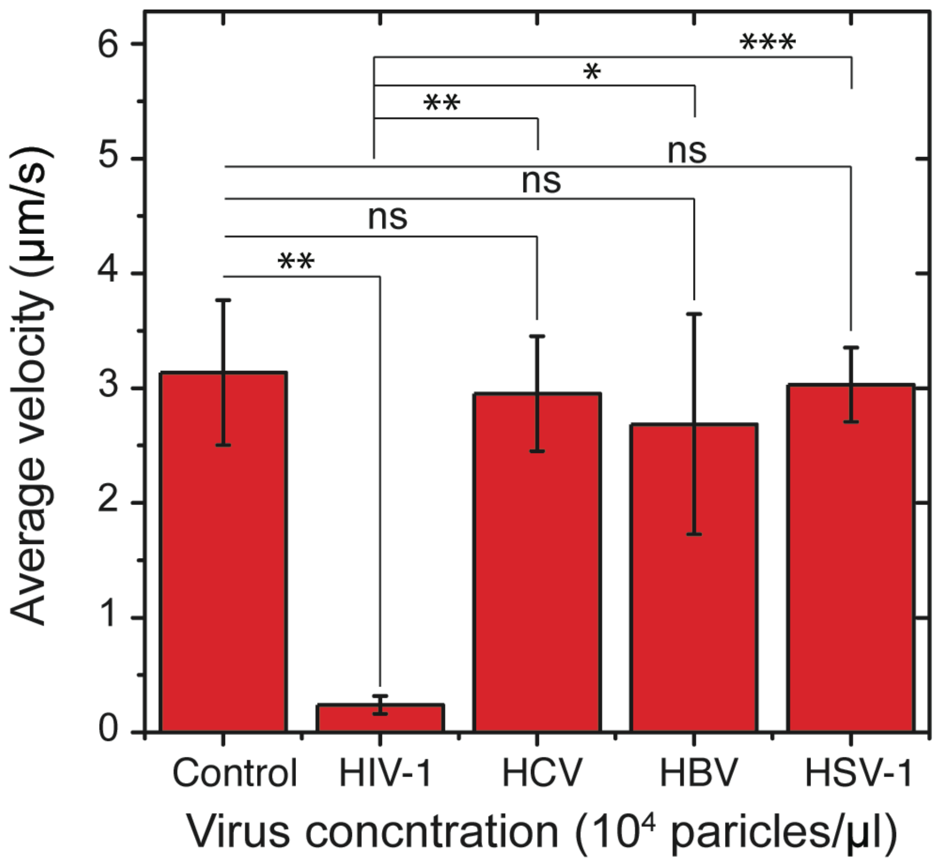

Supplementary Figure 18. Specificity testing of the CALM system using virus-spiked serum samples. Serum samples spiked with the target human immunodeficiency virus 1 (HIV-1) and the non-target viruses of hepatitis C virus (HCV), hepatitis B virus (HBV), and human simplex virus 1 (HSV-1) and control serum samples (no virus) are used to test the detection specificity of the developed CALM system. The results are expressed as the average values of 3 independent experiments. Error bars represent standard deviations. *$P < 0.05$, **$P < 0.01$, ***$P < 0.001$; ns, nonsignificant versus the corresponding control group (no HIV-1), calculated using the unpaired t-test.

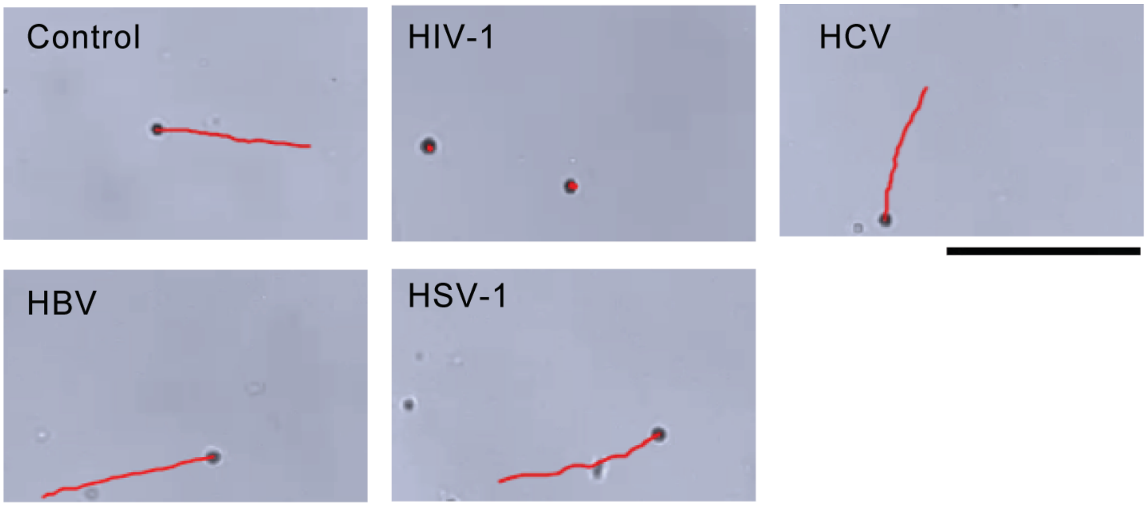

Supplementary Figure 19. Motion of Pt-motors in the presence of target and non-target LAMP amplicons of spiked serum samples. Amplification products generated from target human immunodeficiency virus 1 (HIV-1) and the non-target viruses of hepatitis C virus (HCV), hepatitis B virus (HBV), and human simplex virus 1 (HSV-1) spiked in serum at concentration of 10^4 virus particles/µl. The scale bar is 100 µm.

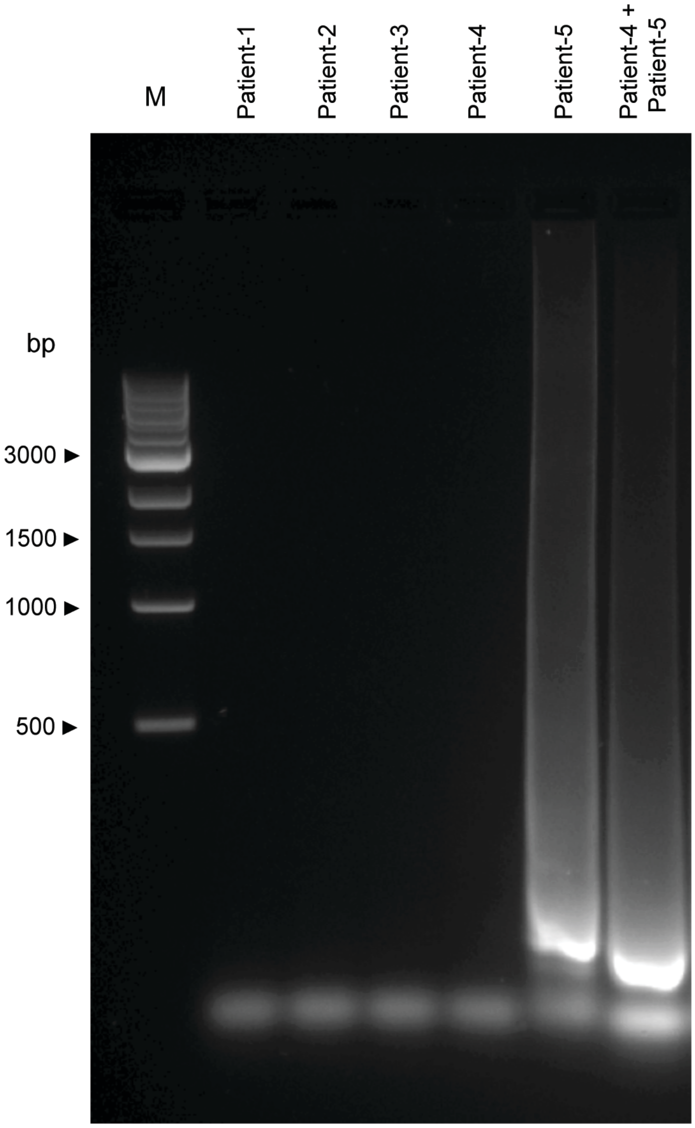

Supplementary Figure 20. Agarose gel electrophoresis image of LAMP amplification products for HIV-1 infected and non-infected patient samples (n = 6).

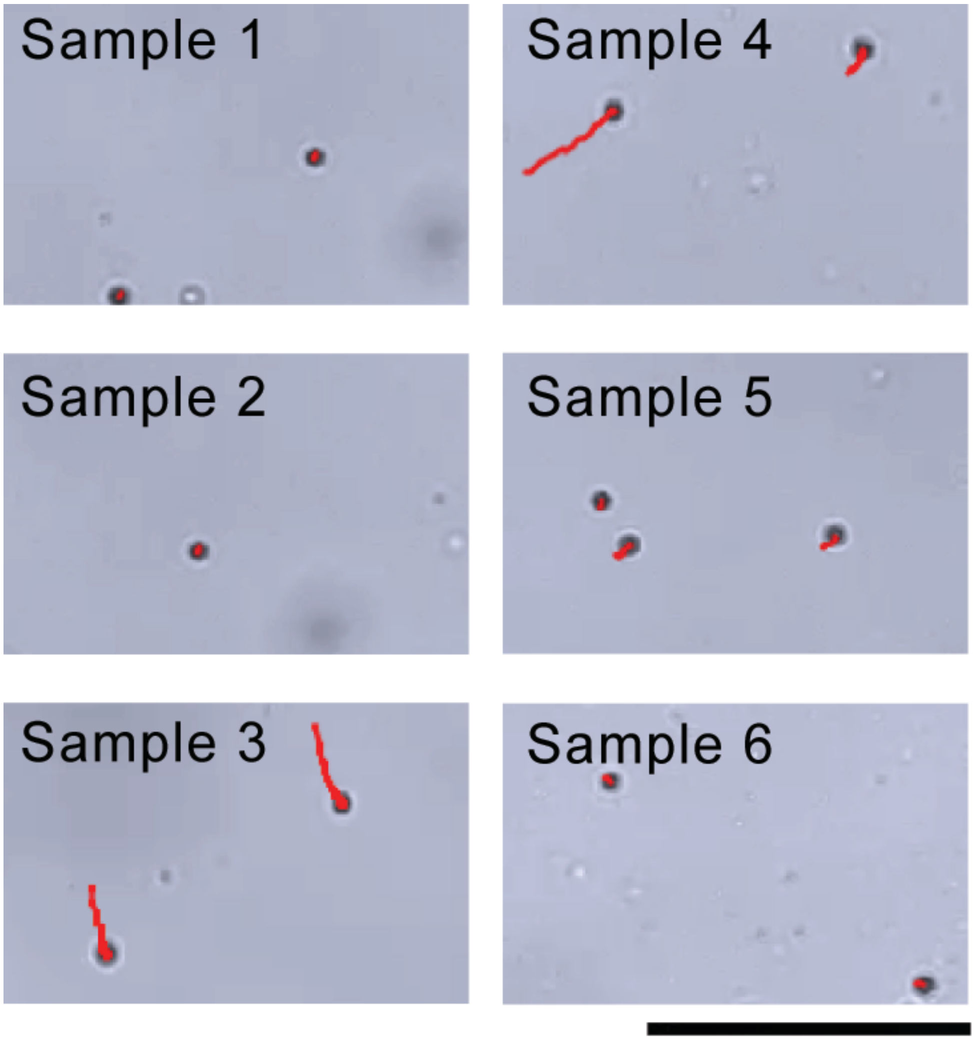

Supplementary Figure 21. Motion of Pt-motors in the presence of LAMP amplification products generated from different HIV-1 infected and non-infected patient samples (n = 6). The scale bar is 100 µm.

Supplementary Table 1. Performance testing of the cellphone system and the bright-field microscopy in Pt-motor counting and motion tracking.

Sample No.	Manual motor count (particle/FOV)[a]	App motor count (particle/FOV)	Manual motor velocity (um/s)[b]	App motor velocity (um/s)
1	0.000	0.00	0	0
2	1.000	1.00	0.05	0.05
3	1.000	1.00	0.05	0.05
4	1.000	1.00	0.05	0.05
5	1.000	1.00	0.05	0.05
6	1.000	1.00	0.05	0.05
7	1.000	1.00	0.186	0.1
8	1.000	1.00	0.106	0.1
9	1.000	1.00	0.15	0.1
10	1.000	1.00	0.203	0.1
11	1.000	1.00	0.11	0.1
12	1.000	1.00	0.117	0.1
13	1.000	1.00	0.1	0.1
14	1.000	1.00	0.184	0.103
15	1.000	1.00	0.103333	0.11
16	1.000	1.00	0.153	0.13
17	1.000	1.00	0.116	0.13
18	1.000	1.00	0.1985	0.19
19	1.000	1.00	0.1985	0.2
20	1.000	1.00	0.237333	0.21
21	1.000	1.00	0.220667	0.23
22	1.000	1.00	0.227	0.23
23	1.000	1.00	0.2885	0.26
24	2.000	1.00	0.397	0.3
25	1.000	1.00	0.328	0.32
26	1.000	1.00	0.675	0.56
27	2.000	2.00	0.603	0.56
28	3.000	2.00	1.25	0.64
29	2.000	2.00	0.65	0.85
30	2.000	2.00	1.06	1.22
31	2.000	2.00	1.412	1.23
32	2.000	2.00	0.69	1.23
33	2.000	2.00	1.17	1.24
34	2.000	2.00	1.687	1.35

[a]the number of motors appeared in the field-of-view (FOV) was manually counted under the cellphone system versus the counts reported by the motion tracking application
[b]the motion of the motors was recorded using bright video microscopy and analyzed using ImageJ software and compared to the motion velocity reported by the motion tracking application

Supplementary Table 1. Continued.

Sample No.	Manual motor count (particle/FOV)	App motor count (particle/FOV)	Manual motor velocity (um/s)	App motor velocity (um/s)
35	2.000	2.00	1.731	1.38
36	2.000	2.00	1.608	1.38
37	2.000	2.00	1.646	1.48
38	2.000	2.00	2.58	1.49
39	2.000	2.00	1.706	1.56
40	2.000	2.00	1.893	1.8
41	3.000	2.00	2.06	2.14
42	4.000	3.00	2.9055	2.15
43	3.000	3.00	2.612	2.43
44	3.000	3.00	3.409	2.83
45	3.000	3.00	3.483	3.39
46	3.000	3.00	3.49	3.55
47	3.000	3.00	2.5	3.55
48	4.000	4.00	3.3565	4.7
49	4.000	4.00	5.37925	4.96
50	4.000	5.00	6.69	6.38

Supplementary Table 2: Sequence of HPV-16 primers used for the LAMP reaction

Name	Sequence (5'-3')
HPV16-F3	TCGGTTGTGCGTACAAAG
HPV16-B3	AGCCTCTACATAAAACCATCC
HPV16-FIP	TGGGGCACACAATTCCTAGTTTTTCACACACGTAGACATTCGT
HPV16-BIP	TCAGAAACCATAATCTACCATGGCTTTTATTACATCCCGTACCC TCTT
HPV16-LF	CCCATTAACAGGTCTTCCAAAGT
HPV16-LB	CCTGCAGGTACCAATGGGG

Supplementary Table 3. Material cost of the 3D printed attachment and the microchip

Item	Cost (USD)
3D printed attachment lenses	1
Polylactic filament	1.2
LED+ Battery+ Wires and switches	1.39
Microchip PMMA	0.175
Microchip DSA	0.035
Glass slides	0.17
RT-LAMP reaction enzymes	0.724
RT-LAMP reaction primers	0.001
RT-LAMP reaction reagents and ultrapure water	0.222
Total	4.917
