# Supplementary material for: DNA engineered micromotors powered by metal nanoparticles for motion based cellphone diagnostics
Source: Nat Commun. 2018 Oct 16;9:4282. doi: 10.1038/s41467-018-06727-8 (PMC6191441; doi:10.1038/s41467-018-06727-8)
Supplement: Supplementary file 16 — Description of Additional Supplementary Information [file 41467_2018_6727_MOESM16_ESM.docx]

Description of Additional Supplementary Files

**Supplementary Movie 1.** The motion of motors in different concentrations of H2O2

**Supplementary Movie 2.** The motion of motors (6 μm) and polystyrene beads (3 μm) in 5% H2O2

**Supplementary Movie 3.** The motion of motors in 5% H2O2 in the presence of HIV-1 LAMP amplicons

**Supplementary Movie 4.** The motion of motors in 5% H2O2 in the absence of HIV-1 LAMP amplicons

**Supplementary Movie 5.** The motion of motors in 5% H2O2 in the presence of 0% HIV-1 LAMP amplicons

**Supplementary Movie 6.** The motion of motors in 5% H2O2 in the presence of 1% HIV-1 LAMP amplicons

**Supplementary Movie 7.** The motion of motors in 5% H2O2 in the presence of 10% HIV-1 LAMP amplicons

**Supplementary Movie 8.** The motion of motors in 5% H2O2 in the presence of 100% HIV-1 LAMP amplicons

**Supplementary Movie 9.** The motion of motors in 5% H2O2 in the presence of LAMP amplicons of virus-free PBS control sample

**Supplementary Movie 10.** The motion of motors in 5% H2O2 in the presence of HIV-1 LAMP amplicons of spiked PBS sample (10000 particles/ml, as positive)

**Supplementary Movie 11.** The motion of motors in 5% H2O2 in the presence of HIV-1 LAMP amplicons of spiked serum sample (100 particles/ml, as negative)

**Supplementary Movie 12.** The motion of motors in 5% H2O2 in the presence of HIV-1 LAMP amplicons of spiked serum sample (5000 particles/ml, as positive)

**Supplementary Movie 13.** The motion of motors in 5% H2O2 in the presence of HIV-1 LAMP amplicons of positive HIV-infected patient sample (>1000 virus particles/ml)

**Supplementary Movie 14.** The motion of motors in 5% H2O2 in the presence of HIV-1 LAMP amplicons of negative HIV-infected patient sample (>1000 virus particles/ml)
